# The Spectrin Cytoskeleton Is Crucial for Adherent and Invasive Bacterial Pathogenesis

**DOI:** 10.1371/journal.pone.0019940

**Published:** 2011-05-16

**Authors:** Tyson Ruetz, Steve Cornick, Julian Andrew Guttman

**Affiliations:** Department of Biological Sciences, Simon Fraser University, Burnaby, British Columbia, Canada; University of Birmingham, United Kingdom

## Abstract

Various enteric bacterial pathogens target the host cell cytoskeletal machinery as a crucial event in their pathogenesis. Despite thorough studies detailing strategies microbes use to exploit these components of the host cell, the role of the spectrin-based cytoskeleton has been largely overlooked. Here we show that the spectrin cytoskeleton is a host system that is hijacked by adherent (Entropathogenic *Escherichia coli* [EPEC]), invasive triggering (*Salmonella enterica* serovar Typhimurium [*S.* Typhimurium]) and invasive zippering (*Listeria monocytogenes*) bacteria. We demonstrate that spectrin cytoskeletal proteins are recruited to EPEC pedestals, *S*. Typhimurium membrane ruffles and *Salmonella* containing vacuoles (SCVs), as well as sites of invasion and comet tail initiation by *L. monocytogenes*. Spectrin was often seen co-localizing with actin filaments at the cell periphery, however a disconnect between the actin and spectrin cytoskeletons was also observed. During infections with *S*. Typhimurium Δ*sipA*, actin-rich membrane ruffles at characteristic sites of bacterial invasion often occurred in the absence of spectrin cytoskeletal proteins. Additionally, early in the formation of *L. monocytogenes* comet tails, spectrin cytoskeletal elements were recruited to the surface of the internalized bacteria independent of actin filaments. Further studies revealed the presence of the spectrin cytoskeleton during SCV and *Listeria* comet tail formation, highlighting novel cytoplasmic roles for the spectrin cytoskeleton. SiRNA targeted against spectrin and the spectrin-associated proteins severely diminished EPEC pedestal formation as well as *S*. Typhimurium and *L. monocytogenes* invasion. Ultimately, these findings identify the spectrin cytoskeleton as a ubiquitous target of enteric bacterial pathogens and indicate that this cytoskeletal system is critical for these infections to progress.

## Introduction

The manipulation of the host cytoskeleton is a crucial step during infections caused by a variety of enteric bacterial pathogens including EPEC, *S.* Typhimurium and *L. monocytogenes*. EPEC attach to host intestinal epithelial cells and remain primarily extracellular during their infections [1]. These microbes utilize a type III secretion system (T3SS) to inject bacterially-derived effector proteins from the bacterial cytosol directly into the host cell cytoplasm [2]. One such effector, the translocated intimin receptor (Tir), is instrumental in anchoring EPEC to the host cell through its extracellular domains. Intracellularly, Tir recruits actin filaments through the binding of actin-related proteins to its cytosolic tail domains. The abundant polymerization of actin filaments beneath EPEC results in the bacteria rising off the natural surface of the cell on actin-rich membrane protrusions called “pedestals”, which are hallmarks of the disease [3], [4].

*S*. Typhimurium also utilize T3SS's as part of their pathogenesis. These invasive pathogens inject a variety of effector proteins, including SopB (SigD), SopE, SopE2 and SipA which cause the host cells to generate intense actin-based membrane ruffles at sites of bacterial invasion [5], [6], [7], [8]. The membrane ruffling engulfs the bacteria into the host cell resulting in their encasement in a vacuole called a *Salmonella* containing vacuole (SCV), providing these microbes a protective niche for replication [9], [10].

*L. monocytogenes*, another invasive pathogen, does not utilize a T3SS but rather deposits its effector proteins on its surface. These bacteria utilize a number of internalin proteins to efficiently enter non-phagocytic host cells; 2 well characterized invasion proteins are internalinA (InlA) and internalinB (InlB)[11]. Both proteins recruit clathrin and the clathrin associated endocytic machinery to sites of bacterial attachment [12], [13]. This collection of proteins initially internalizes the bacterium into a vacuole within the host cytoplasm [14] [15]. Once within the host cell, *L. monocytogenes* quickly disrupts the vacuole that encapsulates it, then initiates the up-regulation and polarized distribution of the ActA effector on the bacterial plasma membrane [16]. ActA mimics N-WASp, thus recruiting the Arp2/3 complex, causing an actin-based comet tail to be generated at one end of the bacterium [16]. This comet tail propels the bacterium within the host cytosol and enables the microbe to disseminate to neighbouring cells [17].

The spectrin cytoskeleton is a well characterised, ubiquitously expressed sub-membranous cytoskeletal system that was first discovered in erythrocytes and has since been identified in a variety of epithelial cells [18], [19], [20]. The cornerstone of this cytoskeletal system is the filamentous polymer spectrin. Unlike other cytoskeletal systems, the spectrin cytoskeleton is thought to be restricted to membranous regions of the cell. Spectrin filaments provide stability and mechanical support to the plasma membrane as well as the Golgi, Golgi associated vesicles, ER and lysosomal membranes of the cell [21], [22], [23]. Spectrin interacts directly with actin filaments as well as the spectrin-associated proteins adducin, protein 4.1 (p4.1) and ankyrin, which provide a bridge between the spectrin-actin cytosketal network and the plasma membrane [24]. Additionally, the spectrin cytoskeleton co-localizes with actin accessory proteins, acting as a “membrane protein-sorting machine” [22] at specific sub-membranous regions of the cell during dynamic membrane remodelling events such as during cell migration [19], [22], [25]. The sub-membranous localization and known actin associations of the spectrin cytoskeleton, together with the dramatic reorganization of the host cell plasma membrane and related cytoskeletal networks during various enteric bacterial infections, suggest that the spectrin cytoskeletal system may also be a target of these pathogens. To examine this, we investigated the role of the spectrin cytoskeleton during EPEC, *S*. Typhimurium and *L. monocytogenes* infections. Our findings show that a set of spectrin cytoskeletal components are targeted by these pathogens and the involvement of this cytoskeletal system is crucial for their pathogenesis.

## Results

### The EPEC effector Tir recruits spectrin, p4.1 and adducin to pedestals

**Figure 1:**
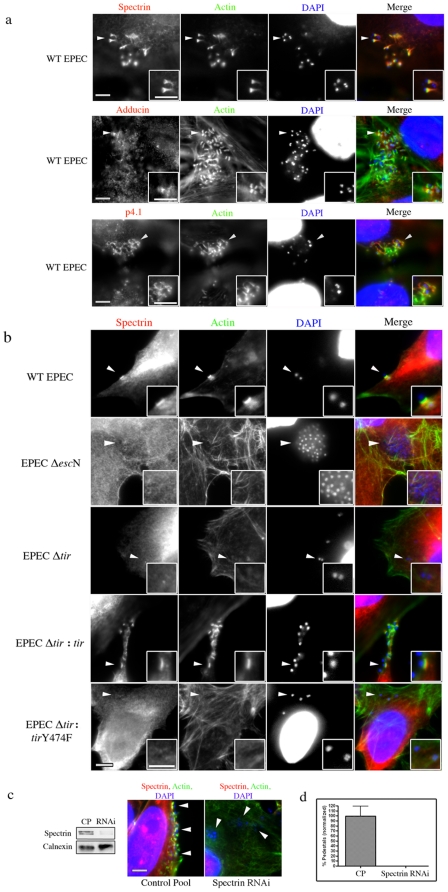
Characterizing the role of the spectrin cytoskeleton during EPEC infections. (**a**) HeLa cells were infected with EPEC and immunolocalized with antibodies to spectrin, adducin and p4.1 together with probes to actin and DAPI. Arrowheads indicate areas of interest that are found in the insets. (**b)** Immunolocalization of spectrin to sites of wild-type (WT) EPEC attachment and to EPEC effector mutants: Δ*escN*, Δ*tir*, Δ*tir*:*tir*, Δ*tir*:*tir*Y474F. (**c)** Western blot of siRNA treated HeLa cells targeted against spectrin (Spectrin RNAi) and non-targeting control pool siRNA (CP). Calnexin was used as a loading control. Immunofluorescent image of spectrin RNAi, attached bacteria show they are unable to form pedestals. (**d**) Quantification of the number of bacteria forming pedestals. For each treatment, 3 independent experiments were run; for microscopy counts n = 3 for each experiment, error bars show s.e.m. Statistics were not run due to a complete absence of pedestals in infected RNAi samples Scale bars are 5 µm.

**Figure 2:**
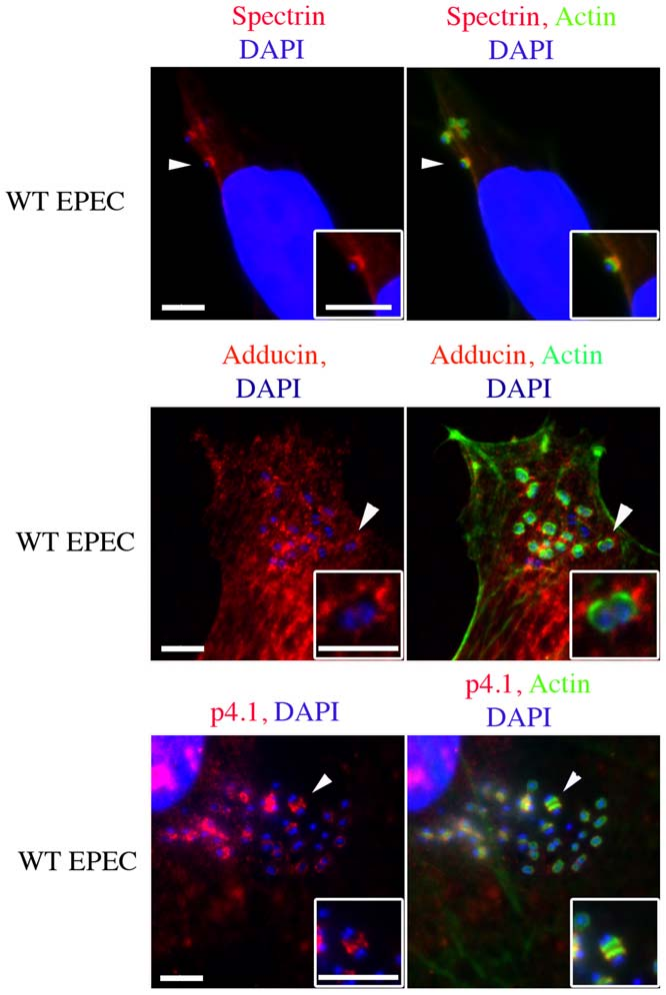
Investigating the precise localization of spectrin cytoskeletal proteins at EPEC pedestals. HeLa cells were infected with EPEC and were immunolocalized with antibodies against spectrin, adducin and p4.1 as well as actin and DAPI. Arrows indicate areas of interest that are found in the insets. Scale bars are 5 µm.

To examine the role of the spectrin cytoskeleton during bacterial infections, we initially infected cultured cells with EPEC and immunolocalized ß_2_-spectrin. We found that spectrin was distinctly recruited to EPEC pedestals, while primary antibody controls showed non-specific staining and no localization at pedestals (Figures 1a HeLa cells, S1 polar Caco cells and S2 controls). To determine whether proteins that are known to interact with spectrin were also present at these sites we immunolocalized the spectrin associated proteins α-adducin and p4.1 and found that they were also present at EPEC pedestals (Figure 1a adducin/p4.1 and S2 controls). When their organization within these structures was analyzed, a slight separation between the bacteria and the spectrin cytoskeleton was observed. Although spectrin-associated proteins co-localized with the actin filaments at certain parts of the pedestals, they were primarily positioned at the basal regions of these structures (Figure 2 and S3).

To determine whether bacterial contact or effector translocation was responsible for spectrin cytoskeletal proteins being concentrated beneath EPEC, we used an EPEC T3SS mutant (EPEC Δ*escN*), mutated in a crucial ATPase needed for effector translocation [26]. Host cells infected with EPEC Δ*escN* did not recruit any components of the spectrin cytoskeleton to sites of bacterial attachment, suggesting that an effector was required (Figures 1b, S4 and S5). Because the EPEC effector Tir is needed for pedestal formation, we examined whether Tir mutants of EPEC concentrated any spectrin-associated proteins at sites of bacterial contact. Infections using EPEC Δ*tir*, did not recruit any components of the spectrin cytoskeleton beneath the bacteria, whereas complemented bacteria (EPEC Δ*tir:tir*) restored the wild-type phenotype (Figure 1b spectrin, S4 adducin and S5 p4.1). Although there are a variety of phosphorylation sites on the EPEC Tir protein that are involved in pedestal formation to varying degrees, by far the most crucial is the tyrosine 474 (Y474) phosphorylation site [27], [28]. To determine whether this site was needed for spectrin cytoskeletal recruitment we used an EPEC Δ*tir* strain complemented with *tir* containing a point mutation at that site (Y474F)(EPEC Δ*tir:tir*Y474F) and examined the localization of spectrin during those infections. Here we again observed a lack of spectrin/adducin/p4.1 recruitment, demonstrating that Tir Y474 phosphorylation is crucial for their positioning during these infections (Figure 1b spectrin, S4 adducin and S5 p4.1). As other EPEC effectors such as EspH, EspZ, Map, EspG and EspF are also proposed to be involved in pedestal formation [29], we examined the recruitment of spectrin/adducin/p4.1 beneath the bacteria during infections with EPEC mutated in each of those effectors and found that in all cases, all three spectrin cytoskeletal proteins were present at pedestals (Figures S6 spectrin, S7 adducin and S8 p4.1).

### Depletion of spectrin cytoskeletal proteins severely impairs EPEC infections

Because the spectrin cytoskeleton appeared to be a significant component of EPEC pedestals, we sought to functionally perturb individual host components to examine their roles in pedestal generation. To accomplish this, we separately transfected HeLa cells with siRNA targeted against β_2_-spectrin, α-adducin and p4.1. Knockdowns were confirmed by western blot analysis (Figures 1c spectrin, S9a adducin and c p4.1). SiRNA pre-treated cells were then infected with wild-type EPEC to examine pedestal formation. In cells with undetectable levels of β_2_-spectrin or p4.1, attached EPEC were unable to form pedestals (Figures 1c, d spectrin, S9d and e p4.1). Despite this, the ability of the bacteria to attach to the host cells was not significantly altered by these treatments (Figure S10). Interestingly, adducin knockdowns resulted in an inability of EPEC to attach to the host cell, thus subsequent pedestal presence was not observed (Figure S9b). To ensure the siRNA treatments were not having adverse effects on the cell, we performed cell viability assays and found no difference in the viability of cells treated with control pool siRNA when compared to spectrin, adducin or p4.1 siRNA treated cells (Figure S11). Furthermore, the actin cytoskeleton of spectrin knocked-down cells was morphologically similar to untreated cells with cortical actin and stress fibers present (Figure S12).

### *S*. Typhimurium usurp the spectrin cytoskeleton during multiple stages of infection

**Figure 3:**
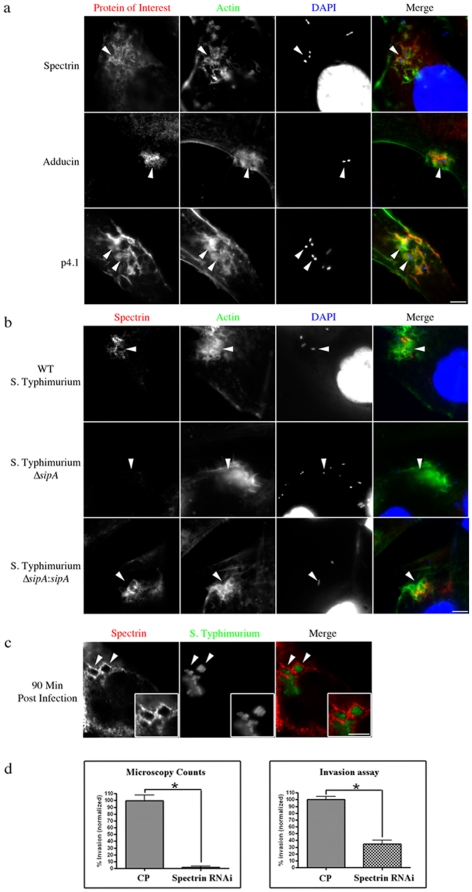
Spectrin cytoskeleton distribution during *S*. Typhimurium infections. (**a**) Immunolocalization of spectrin cytoskeletal elements, spectrin, adducin and p4.1 with actin and DAPI during *S*. Typhimurium SL1344 invasion in HeLa cells. Actin labeling identified invasion sites with actin-rich membrane ruffles. (**b**) Images illustrating the inability of a Δ*sipA* mutant to recruit spectrin to sites of invasion. Δ*sipA*:*sipA* restored spectrin recruitment to invasion sites. (**c**) Spectrin localization to sites of internalized *S*. Typhimurium SL1344-GFP. Areas of interest are indicated by arrowheads and highlighted in insets. (**d**) Quantifying invasion efficiency after depletion of spectrin with siRNA as compared to a non-targeting control pool (CP). For each treatment, 3 independent experiments were run; for microscopy counts n = 3, error bars show s.e.m, *P<0.0001 for all statistics. Scale bars are 5 µm.

Based on our findings with EPEC, we investigated a potential role for the spectrin cytoskeleton during the pathogenesis of another T3SS dependent microbe, *S*. Typhimurium. We found that spectrin was recruited to the actin-rich membranous ruffles at sites of *S.* Typhimurium invasion, but only partially colocalized with actin when examined in detail (Figure 3a HeLa cells, S13 Caco cells and S14 another HeLa cell example). This lack of complete colocalization suggests that the presence of spectrin at these sites was not merely a byproduct of actin recruitment (Figure S13 and S14). The disconnect of actin and spectrin cytoskeletons was confirmed in uninfected cells which showed a lack of spectrin recruitment to a number of stress fibers (Figure S15). In addition to spectrin, the same spectrin associated proteins that were identified at EPEC pedestals (adducin, and p4.1) were also recruited to invasion sites (Figure 3a). To investigate the bacterial factors responsible for this recruitment, we utilized a *S.* Typhimurium Δ*sopE/sopE2/sopB* mutant, deficient in the effectors primarily responsible for membrane ruffling and bacterial invasion during these infections [30]. Infections with this mutant did not generate actin-mediated membrane ruffling and concomitantly the recruitment of the spectrin cytoskeleton to sites of bacterial contact was absent (Figure S16).

*S*. Typhimurium contains the bacterial effector, SipA, which is known to bundle actin and increase efficiency of invasion [31]. To determine if this effector influenced spectrin cytoskeletal protein recruitment to sites of invasion, we immunolocalized spectrin, adducin and p4.1 together with actin during infections with a *S*. Typhimurium *sipA* mutant. Infections with *S*. Typhimurium Δ*sipA* showed that the spectrin and actin cytoskeletons were independently recruited; as actin-rich membrane ruffles remained present but often did not concentrate spectrin or adducin at sites of invasion (Figure 3b spectrin, S17 adducin, and S18 enhanced images). When compared to WT *S*. Typhimurium, *S*. Typhimurium Δ*sipA* invasion sites showed a significantly decreased ability to recruit spectrin and adducin to invasion sites [43% and 89% reduced respectively] (Figure S19). *S*. Typhimurium Δ*sipA* complimented with *sipA* restored the recruitment of spectrin and adducin to the membrane ruffles (Figures 2b spectrin, S17 adducin). P4.1 remained at membrane ruffles irrespective of the presence or absence of SipA (Figures S20).

To investigate the potential involvement of the spectrin cytoskeleton at later time points of infections, when *S*. Typhiurium reside within the SCVs [10], we immunolocalized the spectrin cytoskeletal proteins at 90 minutes post invasion. We found that spectrin, but not adducin or p4.1, was recruited to SCVs (Fig 3c sectrin and S21 adducin/p4.1). We observed distinct localization of spectrin surrounding multiple bacteria within the protective vacuole (Figure 3c). Spectrin, adducin and p4.1 were not observed localizing to bacteria at earlier time points during the intracellular stage of the infections (data not shown).

### RNAi of spectrin, adducin, or p4.1 proteins abolish *S*. Typhimurium invasion

To determine the role of spectrin cytoskeletal components during *S*. Typhimurium invasion, we knocked down individual components of this cytoskeletal system in cultured cells and studied the effects on invasion. Knockdown of spectrin, adducin, or p4.1 proteins in host cells resulted in the near complete cessation of *S*. Typhimurium invasion (Figures 3d spectrin and S22 adducin/p4.1). Quantification of *S*. Typhimurium invasion was assessed by immunofluorescent imaging in which cells were first identified that had undetectable levels of the targeted protein, then the number of bacteria that had infected those cells was counted. Microscopy counts of cells with undetectable levels for each of the three proteins showed an average of 8% invasion compared to control treatments (Figure 3d spectrin and S22 adducin/p4.1). We then quantified invasion efficiencies using classical invasion assay methods. Invasion assays with siRNA pretreated cells resulted in a significant decrease in invasion with an average of 35%/65%/60% (spectrin/adducin/p4.1 RNAi treated) invasion as compared to controls (Figure 3d spectrin and S22 adducin/p4.1). As expected, microscopic analysis showed that our siRNA transfection efficiencies were not %100, with some cells having incomplete knockdown of the targeted protein. The observed increase in invasion efficiencies using the classical invasion assay method as compared to the microscopy-based counts can be attributed to the invasion of unsuccessfully transfected cells and those with only partial knockdowns being present in these assays.

### *Listeria monocytogenes* requires the spectrin cytoskeleton for efficient invasion

**Figure 4:**
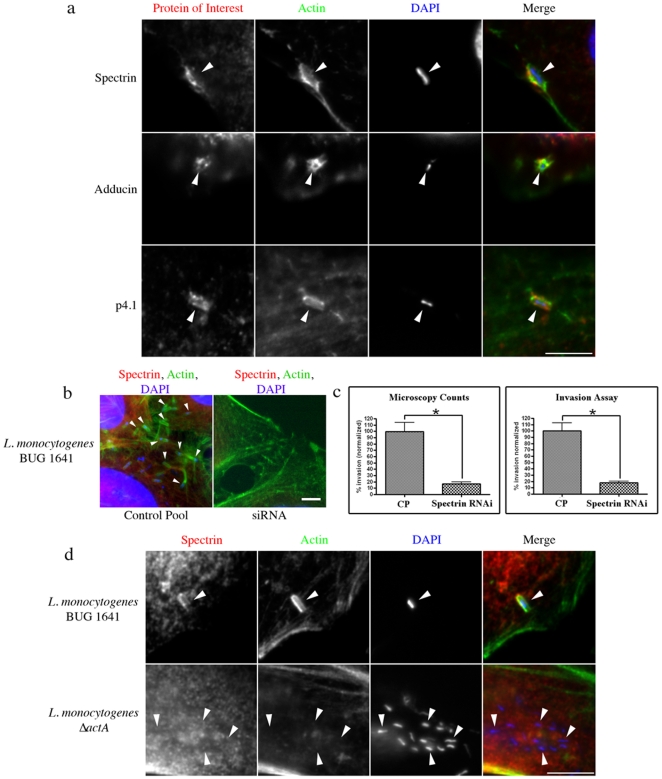
The importance of the spectrin cytoskeleton during *L. monocytogenes* infections. (**a**) Infections showing spectrin/adducin/p4.1 recruitment to the characteristic actin cups at sites of *L. monocytogenes* invasion. (**b**) Immunofluorescent image of spectrin RNAi showing inability of *L. monocytogenes* to invade cells with no detectable spectrin. Arrows indicate internalized bacteria. (**c**) Graphs quantifying invasion efficiency after depletion of spectrin with siRNA as compared to control pool (CP) siRNA treatment. For each treatment, 3 independent experiments were run, for microscopy counts n = 3, error bars show s.e.m, *P<0.0001 for all statistics. (**d**) Initial stages of *L. monocytogenes* comet tail formation, showing polar spectrin recruitment together with actin. Infection with *Listeria* Δ*actA* did not recruit spectrin. Scale bars are 5 µm.

We further characterized the role of the spectrin cytoskeleton during bacterial invasion by studying *L. monocytogenes* infections. Infections of cultured cells, which allow only the InlB invasion pathway to ensue [12], showed spectrin/adducin/p4.1 lining the characteristic actin-rich sites of *L. monocytogenes* internalization (Figure 4a) [32]. Individual siRNA-based depletion of spectrin/adducin/p4.1 nearly abolished the ability of *L. monocytogenes* to invade the host cell (Figure 4b and S24). Microscopy counts of cells with undetectable levels of spectrin/adducin/p4.1 showed 17%/15%/6% invasion compared to control treatments (Fig 4c spectrin and S24 adducin/p4.1). Classical invasion assays performed on these samples (which include unsuccessfully transfected cells) resulted in significantly different levels of invasion when RNAi treated cells were compared to controls [21%/52%/60% spectrin/adducin/p4.1 respectively] (Figures 4c spectrin and S24 adducin/p4.1).

### *L. monocytogenes* recruits Spectrin and p4.1 to initial stages of comet tail formation using the ActA effector

**Figure 5:**
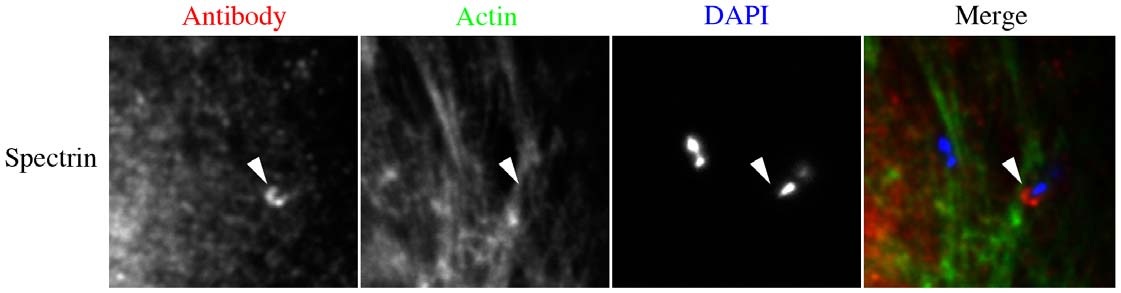
Spectrin is recruited to initial stages of *L. monocytogenes* tail formation, independent of actin. Immunolocalization of spectrin to internalized *L. monocytogenes* at 30 minutes post infection. Scale bars are 5 µm.

Following entry into host cells, *L*. *monocytogenes* up-regulate the ActA effector to initiate the formation of the characteristic actin-rich comet tails [33]. We found that spectrin and p4.1 were recruited to the initial stages of comet tail formation, whereas adducin was not (Figure 4d spectrin in HeLa cells, S23 spectrin in polar Caco cells and S25 adducin/p4.1 in HeLa cells). Detailed analysis revealed that in some instances spectrin was localized to the bacteria independent of actin (Figure 5 HeLa cells and S23 Caco cells). At 30 minutes post infection, 70% of the bacteria had spectrin lining the membrane in the absence of actin, whereas after 90 minutes of infection only 7% of internalized bacteria were associated with spectrin alone (Figure S26). Infections with *L. monocytogenes* ActA mutants (*L. monocytogenes* Δ*actA*) resulted in the absence of spectrin and p4.1 with the internalized bacteria, suggesting that ActA is needed for their recruitment (Figures 4d spectrin and S25 p4.1). Upon mature, full-length comet tail formation, spectrin as well as adducin and p4.1 were absent (Figure S27).

## Discussion

In this study we have shown that a set of spectrin cytoskeletal proteins are co-opted during a variety of enteric bacterial infections. We have demonstrated that spectrin, adducin and p4.1 are crucial proteins involved in EPEC pedestal formation, *S.* Typhimurium and *L. monocytogenes* epithelial cell invasion and subsequent stages of their intracellular life cycles. By functionally perturbing these host proteins, infections were efficiently halted demonstrating that this cytoskeletal system is integral to the pathogenesis of these bacteria.

During our examination of EPEC infections we showed that spectrin was specifically concentrated at the base of pedestals, partially colocalizing with actin. This basal localization resembles that of other membrane protruding structures, namely microvilli and filopodia. Such structures contain a spectrin-based scaffold that provides a secure foundation for protein machinery localization, thus enabling the remodeling of the plasma membrane [25], [34]. Consequently, spectrin may be providing a similar function during EPEC pedestal formation, by providing a substratum for membrane protrusion and pedestal formation. This is supported by evidence demonstrating that attached EPEC were unable to recruit actin beneath the bacteria when any of the spectrin cytoskeletal components were knocked-down.

*S*. Typhimurium internalization is heavily dependant on actin-based membrane ruffles, however evidence presented here demonstrates that spectrin cytoskeletal components are also needed for maximal invasion. When any of the three spectrin cytoskeletal proteins were knocked-down, we observed ∼8% invasion efficiencies when invaded bacteria were counted by microscopy in cells with undetectable levels of those cytoskeletal components. *S*. Typhimurium use a multitude of effector proteins to efficiently invade non-phagocytic cells. During infections with *S*. Typhimurium mutated in SipA, an effector involved in actin bundling that is known to aid in invasion [8], we found that actin-rich membrane ruffles remained present but often lacked spectrin or adducin. Those results suggested that the presence of SipA was required for the efficient targeting of those 2 components to the ruffles. Others have shown that infections using *S*. Typhimurium Δ*sipA* resulted in ∼60% invasion efficiency compared to wild-type infections [8]. Our classical invasion assay results demonstrated similar invasion efficiencies when spectrin cytoskeletal components were knocked-down. Taken together these results support an important role for the SipA effector in spectrin/adducin recruitment and suggest that *S*. Typhimurium posses strategies to control the spectrin cytoskeleton independently of the actin cytoskeleton.

*L. monocytogenes* utilize clathrin-mediated endocytosis (CME) to gain entry into non-phagocytic cells [12], [13]. The involvement of the spectrin cytoskeleton during CME has been examined by others and shown to be excluded from clathrin-coated pits to encourage budding from the plasma membrane [23], [35], [36]. Based on this, we expected that spectrin would be absent from *L. monocytogenes* invasion sites in a similar fashion to classical CME. However, we found that spectrin was recruited to sites of *L. monocytogenes* invasion. Furthermore, when we knocked-down spectrin using siRNA, infections were inhibited; demonstrating that spectrin is needed for clathrin mediated *L. monocytogenes* uptake. Although entry of *L. monocytogenes* into epithelial cells involves the internalization of a structure that is large in comparison to a classically formed endocytic particle [12], [13], our results contradict the traditional views of spectrin's role in CME and require further scrutiny.

The spectrin cytoskeleton has been extensively characterized as a network restricted to the eukaryotic plasma membrane and membrane domains of the Golgi, Golgi associated vesicles, ER and lysosomes [19], [37]. Accordingly, we anticipated that internalized bacteria found within the host cell cytosol would not associate with the spectrin cytoskeleton. However, we observed that after internalization, *L. monocytogenes* were able to recruit spectrin and p4.1 to sites of initial comet tail formation suggesting that this cytoskeletal system is not restricted to membranous regions of eukaryotic cells as previously thought.

Clues to understanding the function of spectrin during *L. monocytogenes* comet tail formation may lie in other systems. During cell migration, spectrin associates with actin machinery to facilitate actin polymerization for subsequent motility [22], [25]. Although this potential function provides a likely role for spectrin during *L. monocytogenes* infections, we were unable to directly investigate bacterial motility in the absence of spectrin cytoskeletal components due to the severe defects of *L. monocytogenes* invasion in cells knocked-down in any of the spectrin cytoskeletal proteins. Despite this we were able to determine whether spectrin cytoskeletal components required any bacterial surface protein for their recruitment to the bacteria. During, infections with *L. monocytogenes* Δ*actA*, the bacteria were able to invade cells but were unable to recruit spectrin and p4.1 to internalized bacteria, suggesting that the spectrin cytoskeleton was not simply recruited to the bacterial membrane, but required the presence of the ActA effector to initiate its recruitment at the bacteria for subsequent comet tail formation.

Although our findings have demonstrated an integral role for the spectrin cytoskeleton during a variety of pathogenic infections our findings have opened the door to many important questions that will require future examination. First will be to investigate the crucial domains of spectrin, adducin and p4.1 that are responsible for their recruitment to sites of infection. In addition to this, further exploration into the dynamics of spectrin cytoskeletal protein recruitment in relation to actin cytoskeletal components during these infections is required. Finally, understanding how the depression of adducin expression interferes with EPEC binding to the host cells and mechanistically elucidating the precise influence that SipA has on the spectrin cytoskeleton during *S*. Typhimurium infections will require further scrutiny.

Ultimately, our identification of the spectrin cytoskeleton as a target during key stages of adherent, triggering and zippering enteric bacterial pathogenesis, demonstrates that this previously overlooked cytoskeletal system is integral to a variety of infections. This recruitment, coupled with the demonstration that the depletion of spectrin cytoskeletal proteins from host cells during these infections results in the inhibition of bacterial attachment and invasion, highlights the importance of this cytoskeletal system in disease progression. Accordingly, the broad involvement of the spectrin cytoskeleton with enteric microbial pathogens reveals a new potential target for therapeutic treatments of these infections.

## Materials and Methods

### Cells, Bacteria, and Growth Conditions

Cells (from ATCC) were grown in Dulbecco's Modified Eagle Medium (DMEM) (Hyclone) supplemented with 10% (20% for Caco-2 cells) Fetal Bovine Serum (FBS) (Sigma). HeLa cells are a commonly used cell line for invasion and pedestal research [12], [13], [38], [39], [40]. We opted to use them due to their flat morphology and ease of imaging. The bacterial strains used in this study included wild-type Enteropathogenic *E.coli* (EPEC) strain E2348/69, EPEC Δ*escN*, EPEC (strain JPN15) and mutants from the same strain including Δ*tir*, Δ*tir* complimented with EPEC *tir*, and Δ*tir* complemented with EPEC *tir* Y474F (JPN15 Δ*tir*+*tir*ΔY474), wild-type Salmonella Typhimurium (strain SL1344) S. Typhimurium SL1344 mutants (Δ*sipA*, Δ*sopB*(*sigD*)/*sopE*/*sopE2*, and Δ*sipA*:*sipA*), S. Typhimurium SL1344 GFP, wild type *L. monocytogenes* (strain EGD600) and *L. monocytogenes* mutants Δ*actA* (strain 2140) and a hyperinvasive strain, expressing an *inlB* derivative containing an NH2 terminal region (reference the paper where this was used). All EPEC and S. Typhimurium strains were grown using standard luria broth (LB), and *L. monocytogenes* was grown using brain heart infusion (BHI) agar (BD Biosciences), including antibiotics where appropriate.

Caco-2 human colon epithelial cells were polarized using the BIOCOAT® HTS Caco-2 Assay System as per manufacturers instructions (BD Biosciences). Briefly, cells were grown to 100% confluency and maintained for 2 days prior to seeding on 1.0 µm fibrillar collagen coated PET membranes. Seeding was performed in the seeding basal medium provided, then replaced 24 hours later by the Entero-STIM Medium provided. All media was supplemented with the provided MITO+serum extender. After 48 hours the cells established a polarized monolayer [41]. At this point, the media was replaced with DMEM (with 10% FBS), and the cells were used for experiments.

### Infections

For HeLa cell infections, cells were grown to approximately 70% confluency, whereas Caco-2 cells were fully confluent. Following overnight cultures, EPEC was used to infect host cells at a multiplicity of infection (MOI) of 10∶1 for 6 hours and followed procedures previously described [42]. For S. Typhimurium studies of initial invasion, subcultures of overnight bacteria were back-diluted 30X in fresh LB and grown at 37°C (shaking) for 3 hours to activate the *Salmonella*, cells were infected at an MOI of 100∶1 and the infections were carried out for 15 minutes. For *L. monocytogenes* studies, overnight bacterial cultures were diluted 10X, then cultured until A_600 nm_ = 0.8. The cells were then infected at an MOI of 50∶1. For initial invasion studies, we infected the cells for 15 minutes prior to fixation, whereas for comet tail studies infections persisted for 30 minutes at which point the media was swapped with warm media containing gentamicin for 1 hour (initial comet tail formation) or 4 hours (established comet tail studies).

### Invasion Assays

To perform invasion assays *L. monocytogenes* or *S*. Typhimurium were incubated on host cells for 30 minutes. This was followed by a 1-hour incubation in media containing 50 µg/ml gentamicin (to kill external bacteria). Cells were then washed 5 times in PBS (Supplemented with magnesium and calcium; Hyclone), and then permeabilized with 1% triton for 5 minutes. Serial dilutions were then prepared, spread on LB plates and incubated for 24 hours at 37°C prior to enumeration.

### Antibodies and Reagents

Antibodies used in this study included a mouse monoclonal anti-β-Spectrin II antibody (used at 2.5 µg/ml for immunofluorescence and 0.25 µg/ml for western blots) (Becton Dickinson), rabbit anti-α-adducin (used at 2 µg/ml for immunofluorescence and 0.2 µg/ml for western blots) (Santa Cruz), rabbit anti-EPB41 (protein 4.1) (used at 1.7 µg/ml for immunofluorescence and 0.17 µg/ml for western blots) (Sigma), rabbit anti-calnexin (Becton Dickinson) (used at 1∶2000). Secondary antibodies included a goat anti-mouse (or rabbit) antibody conjugated to AlexaFluor 568/594 (use at 0.02 µg/ml) (or HRP used at 1 µg/ml for western blotting) (Invitrogen). For F-actin staining AlexaFluor 488 conjugated phalloidin (Invitrogen) was used according to the manufacturers instructions.

### Immunofluorescent Localizations

Cells were fixed on cover slips with 3% paraformaldehyde for 15 minutes at room temperature, permeabilized using 0.1% Triton for 5 minutes at room temperature, then washed 3 times (10 minutes each) with PBS -/- (Hyclone). Samples were blocked in 5% normal goat serum in TPBS/0.1% BSA (0.05% Tween-20 and 0.1% BSA in PBS) for 20 minutes. Antibodies were then incubated on the cover slips overnight at 4°C. The next day the cover slips were washed three times (10 minutes each) with TPBS/0.1% BSA. After the final wash, secondary antibodies were applied for 1 hr at 37°C. This was followed by three additional washes (10 minutes each) with TPBS/0.1% BSA. The cover slips were then mounted on slides using Prolong Gold with DAPI (Invitrogen).

### Transfection of siRNA

β-Spectrin II, protein 4.1, α-adducin and a control pool of siRNA (Dharmacon) were transfected using the InterferIN transfection reagent (PolyPlus Transfection) according to the manufactures instructions. Transfections were incubated for 48 hours. The media was changed prior to the infections.

### Western Blots for RNAi confirmation

Infections were performed as described above. Following the infections, the samples were placed on ice and 120 µl of ice-cold RIPA lysis buffer (150 mM NaCl, 1 M Tris pH 7.4, 0.5 M EDTA, 1% Nonidet P-40, 1% Deoxychloric acid, 0.1% SDS) with EDTA Free COMPLETE protease inhibitors (Roche). Protein lysate concentrations were determined using a bicinchoninic acid assay. The samples were processed and loaded into 6% (or 10% for Adducin and protein 4.1) poly-acrylamide gels and were run at 100 V. The proteins were then transferred to nitrocellulose membranes (Trans-Blot transfer medium, Bio-Rad). Membranes were blocked with 5% Blotto (Santa Cruz Biotechnology) for 20 minutes prior to incubation with primary antibodies (for concentrations see ‘Antibodies and Reagents’ section) overnight at 4°C. Blots were then washed three times with TPBS-BSA (1% Tween-20 in PBS, with 0.1% BSA) then incubated with HRP (at 1 µg/ml) for five minutes and visualized using chemiluminescence BioMax film (Kodak). Blots were then stripped (with 2%SDS, 12.5% Tris pH 6.8, 0.8% β-mercaptoethanol) for 45 minutes at 50°C, re-probed with antibodies used for loading controls and visualized by chemiluminescence.

### Quantifying bacterial pathogenic events during siRNA knockdowns using microscopy

Quantification of EPEC, *S.* Typhimurium and *L. monocytogenes* experiments in which specific proteins were knocked down by siRNA in host cells were performed by initially identifying cells with undetectable levels of the knocked down proteins (spectrin, adducin or p4.1). After identifying these cells, we manually counted the number of bacteria that had successfully generated pedestals (EPEC) or invaded (S. Typhimurium and *L. monocytogenes*) those cells.

### Cell viability assays for siRNA treated cells

Cell viability assays performed on siRNA treated (or untreated) cells were performed using the LIVE/DEAD® Cell Viability Assay kit (Invitrogen), as per manufacturers instructions.

### Controls

Primary antibody controls were performed by replacing the primary antibody with normal mouse IgG (Jackson ImmunoResearch) at the identical concentration to what the primary antibody was used at. Secondary antibody controls were performed by replacing the primary antibody with TPBS/0.1%BSA (the carrier buffer for the primary antibodies), while all other procedures remained unchanged. We tested for autofluorecence in cells and bacteria by replacing the primary and secondary antibodies with buffer and then mounting the cover slips with Prolong Gold (with Dapi).

### Statistics

Statistical analysis to compare the means of two samples, comprised an un-paired, single tailed, student t-tests, with *P* values as indicated.

## Supporting Information

Figure S1 Spectrin is recruited to EPEC pedestals on polarized Caco-2 cells. Polar Caco-2 monolayers were infected with EPEC and stained for spectrin, actin and DAPI. Arrow points to area of actin and spectrin recruitment that is magnified within the inset. Scale bars are 5 µm.
(TIF)

Figure S2 Primary antibody controls show no specific staining at EPEC pedestals. HeLa cells were infected with EPEC for 6 hours. Cells were treated with antibodies specific to spectrin or p4.1 and compared to cells stained with normal mouse IgG (NMsIgG) or normal rabbit IgG (NRbIgG), at identical concentrations to the spectrin and p4.1 antibodies respectively. Primary antibodies or non-specific IgG were co-localized with probes for DAPI and actin to identify attached EPEC and their pedestals. Scale bars are 5 µm.
(TIF)

Figure S3 Spectrin localizes to the basal region of EPEC pedestals. HeLa cells were infected with EPEC and stained for spectrin and actin. Arrows indicate a concentration of spectrin at the pedestal but it is not recruited to areas of actin filament concentration.
(TIF)

Figure S4 The role of EPEC effectors in adducin recruitment to pedestals. HeLa cells were infected with EPEC or EPEC effector mutants, and immunolocalized with adducin antibodies, as well as actin and DAPI. Arrows indicate areas of interest that are found in the insets. Images examining adducin localization in uninfected (UI) or infections with WT EPEC, EPEC Δ*esc*N, EPEC Δ*tir*, EPEC Δ*tir*:*tir*, and EPEC Δ*tir*:*tir*Y474F. Scale bars are 5 µm.
(TIF)

Figure S5 P4.1 recruitment in host cells during EPEC infections. HeLa cells were infected with various EPEC effector mutants and immunolocalized with an antibody targeted against p4.1, as well as probes to actin and DAPI. Arrows indicate areas of interest that are found in the insets. Figure shows immunolocalization of p4.1 during infections with WT EPEC, EPEC Δ*esc*N, EPEC Δ*tir*, EPEC Δ*tir*:*tir*, EPEC Δ*tir*:*tir*Y474F. Scale bars are 5 µm.
(TIF)

Figure S6 Spectrin recruitment to pedestals generated during other EPEC effector mutants that are also involved in efficient pedestal formation. HeLa cells were infected with EPEC or EPEC effector mutants and immunolocalized with an anti-spectrin antibody and co-localized with actin and DAPI. Figure shows immunolocalization of spectrin during infections with WT EPEC, EPEC Δ*espH*, EPEC Δ*espZ*, EPEC Δ*map*, EPEC Δ*espG*, EPEC Δ*espF.* Scale bars are 5 µm.
(TIF)

Figure S7 Immunolocalization of adducin, actin and DAPI during infections with EPEC effector mutants on HeLa cells. The figure shows immunolocalization of adducin to pedestals of WT EPEC, EPEC Δ*espH*, EPEC Δ*espZ*, EPEC Δ*map*, EPEC Δ*espG*, EPEC Δ*espF.* Scale bars are 5 µm.
(TIF)

Figure S8 P4.1 actin and DAPI co-localization during infections with EPEC effector mutants. Figure showing recruitment of p4.1 to pedestals of WT EPEC, EPEC Δ*espH*, EPEC Δ*espZ*, EPEC Δ*map*, EPEC Δ*espG*, EPEC Δ*espF.* Scale bars are 5 µm.
(TIF)

Figure S9 Adducin and p4.1 are crucial for EPEC attachment and pedestal formation repectively. (**a**) Adducin was knocked-down in host cells. (**b**) EPEC infected cells were labeled with adducin, actin and DAPI. Bacteria did not attach to adducin RNAi cells, but attached and generated pedestals in cells with no treatment (NT) and control pool (CP) siRNA treated cells. (**c**) Western blot confirming p4.1 was knocked down using siRNA (RNAi). Cells were infected with wild-type EPEC and pedestals counted. (**d**) Immunofluorescent images and (**e)** quantification of the number of bacteria forming pedestals. For each treatment, 3 independent experiments were run; for microscopy counts n = 3, error bars show s.e.m. No stats run due to a complete absence of pedestals generated in infected RNAi samples. Scale bars are 5 µm.
(TIF)

Figure S10 Spectrin or p4.1 knockdowns do not influence the ability of EPEC to attach to the host cell. HeLa cells were transfected with control pool (CP), spectrin or p4.1 siRNA, then infected with EPEC for 6 hours. The average number of bacteria attached to each cell was then counted. Each experiment was run in triplicate (n = 3) and 30 host cells were counted per treatment. The means of each treatment were not statistically significant (P<0.05). Error bars show s.e.m.
(TIF)

Figure S11 Viability of cells is unaltered by various siRNA treatments. Hela cells were left untreated (NT = no treatment) or treated with control pool (CP), spectrin, p4.1, or adducin siRNA identically to our infection siRNA protocols. (a) The cells were stained with a cell viability probe (Invitrogen). Green cells represent viable cells, red cells represent dead cells. For each treatment, 3 independent experiments were run (n = 3). (b) Total cell viability of each treatment was quantified by counting 200 cells in each sample. The means of each treatment are not statistically significant (P<0.05). Error bars show s.e.m. Scale bar is 5 µm.
(TIF)

Figure S12 Actin cytoskelton morphology is unaltered during spectrin knockdown. HeLa cells were treated with spectrin siRNA for 48 hours. Cells were stained for actin, spectrin and DAPI. The actin cytoskeleton morphology appears normal, with characteristic cortical actin and stress fibers present in the cells. Scale bar is 5 µm.
(TIF)

Figure S13 Spectrin is recruited to membrane ruffles during *S.* Typhimurium invasion of Caco-2 cell monolayers. Polarized Caco-2 cells were infected with *S.* Typhimurium for 15 minutes and immunolocalized with spectrin, actin and DAPI. Arrows indicated regions where spectrin is present peripheral to actin at the membrane ruffles. Scale bar is 5 µm.
(TIF)

Figure S14 Spectrin is present at regions of *S.* Typhimurium membrane ruffles independent of actin. Immunolocalization of spectrin, actin and DAPI during infection of HeLa cells with *S.* Typhimurium. Arrowhead and inset identify a site of invasion, demonstrating spectrin recruitment at site of bacterial invasion that are independent of actin in certain regions. Scale bars are 5 µm.
(TIF)

Figure S15 Examples of actin cytoskeletal network in regions where spectrin is absent in uninfected cells. Uninfected HeLa cells were stained for actin, spectrin, and DAPI. Stress fibers and the cell cortex are present with actin in the absence of spectrin. Scale bar is 5 µm.
(TIF)

Figure S16 Membrane ruffles are needed for spectrin cytoskeletal protein recruitment. Immunofluorescence images of spectrin, adducin and p4.1 with DAPI and actin during infection with *Salmonella* Δ*sopE/E2/B* mutant compared to WT Salmonella. Arrows indicate areas of interest. *S*. Typhimurium Δ*sopE/E2/B* did not generate membrane ruffles and did not recruit spectrin cytoskeletal proteins. Scale bar is 5 µm.
(TIF)

Figure S17 SipA is required for adducin recruitment to membrane ruffles. Adducin was immunolocalized during *S*. Typhimurium Δ*sipA* infections. Images show a lack of adducin recruitment to invasion sites with actin-rich membrane ruffling on HeLa cells infected with *S*. Typhimurium Δ*sipA*. Complemented *S*. Typhimurium Δ*sipA:sipA* rescued the wild-type phenotype. Scale bar is 5 µm.
(TIF)

Figure S18 *S.* Typhimurium Δ*sipA* infections with over-enhanced images to indicate that a cell was present in the S. Typhimurium Δ*sipA* panels in figures 3b and S15. Background spectrin and adducin host cell levels are presented. Scale bars are 5 µm.
(TIF)

Figure S19 *S*. Typhimurium Δ*sipA* infections showed reduced ability to recruit spectrin and adducin. Quantification of spectrin and adducin recruitment to sites of *S*. Typhimurium Δ*sipA* invasion as compared to WT *S*. Typhimurium invasion. Invasion sites were identified by actin-rich membrane ruffles around attached bacteria, then observed for spectrin or adducin recruitment to those sites. Each experiment was performed in triplicate (n = 3), counting 100 actin-based invasion sites. The means of the WT versus Δ*sipA* infection are significant (P<0.0001). Error bars show s.e.m.
(TIF)

Figure S20 P4.1 accumulation is unaltered by a mutation in *sipA*. *S*. Typhimurium Δ*sipA* infected HeLa cells recruited protein 4.1 to membrane ruffles during invasion. P4.1 localization was maintained during *S*. Typhimurium wild-type, Δ*sipA,* or Δ*sipA:sipA* infected cells. Scale bar is 5 µm.
(TIF)

Figure S21 Adducin and p4.1 are not recruited to SCV's. *S*. Typhimurium infected HeLa cells were immunolocalized with anti-adducin or anti-p4.1 antibodies together with DAPI at 90 minutes post infection. No accumulation of adducin or protein 4.1 was detected. Areas of interest are indicated by arrowheads and highlighted in insets. Scale bars are 5 µm.
(TIF)

Figure S22 Adducin and p4.1 are crucial for efficient *S*. Typhimurium invasion. Adducin and p4.1 were individually knocked down in HeLa cells and infected with wild-type *S*. Typhimurium. Samples were assayed my microscopic counts and invasion assays and compared to non-targeting control pools (CP) of siRNAs. For each treatment, 3 independent experiments were run; for microscopy counts n = 3, error bars show s.e.m, *P<0.0001 for all statistics. Microscopy counts focused on cells with complete knockdown, counting total number of internalized bacteria. Invasion assays involved typical gentamicin survival assay.
(TIF)

Figure S23 Spectrin is recruited to *L. monocytogenes* at the initial stages of comet tail formation in polar Caco-2 cells. Caco-2 monolayers were infected with *L. monocytogenes* for 30 minutes and stained for spectrin, actin and DAPI. Large arrows show areas where spectrin was recruited to bacterial membranes without actin, while small arrows show co-localization of spectrin and actin at the bacterial membrane. Scale bar is 5 µm.
(TIF)

Figure S24 Adducin and p4.1 are crucial for efficient Listeria invasion. Adducin, p4.1 or control pool (CP) non-targetting RNAi treated HeLa cells were infected with *L. monocytogenes* and quantified by microscopy and invasion assays. For each treatment, 3 independent experiments were run; for microscopy counts n = 3, error bars show s.e.m, *P<0.0001 for all statistics. Microscopy counts focused on cells with complete knockdown, counting total number of internalized bacteria. Invasion assays involved typical gentamicin survival assay.
(TIF)

Figure S25 Act A is needed for p4.1 recruitment to sites of *L. monocytogenes* comet tail formation but does not influence the lack of adducin recruitment. Immunoflourescence images depicting p4.1, adducin, actin and DAPI at 90 minutes post infection. *L. monocytogenes* Δ*actA* infections show no actin, adducin or p4.1 recruitment, whereas wild-type *L. monocytogenes (*BUG 1641*)* containing *actA* recruits actin and p4.1, but not adducin. Scale bars are 5 µm.
(TIF)

Figure S26 Quantification of spectrin localized at internalized *L. monocytogenes* in the absence of actin. HeLa cells infected for 30 or 90 minutes with *L. monocytogenes* were immunolocalized with spectrin, actin and DAPI. Internalized bacteria associated with spectrin were quantified and compared to bacteria associated with both spectrin and actin. The graph depicts the percentage of bacteria associated with spectrin alone at various time points. 100 internalized bacteria were counted per experiment (n = 3). Each experiment was run in triplicate. The means of the two data sets are significantly different (P<0.05). Error bars show s.e.m.
(TIF)

Figure S27 Spectin cytoskeletal components are absent from established *L. monocytogenes* comet tails. Spectin, adducin, and p4.1 together with actin and DAPI were labeled on *L. monocytogenes* infected HeLa cells 3 hours post infection. None of the spectrin cytoskeletal proteins were recruited to comet tails. Scale bars are 5 µm.
(TIF)
