# Supplementary figures and images for: The Spectrin Cytoskeleton Is Crucial for Adherent and Invasive Bacterial Pathogenesis
Source: PLoS One. 2011 May 16;6(5):e19940. doi: 10.1371/journal.pone.0019940 (PMC3095645; doi:10.1371/journal.pone.0019940)

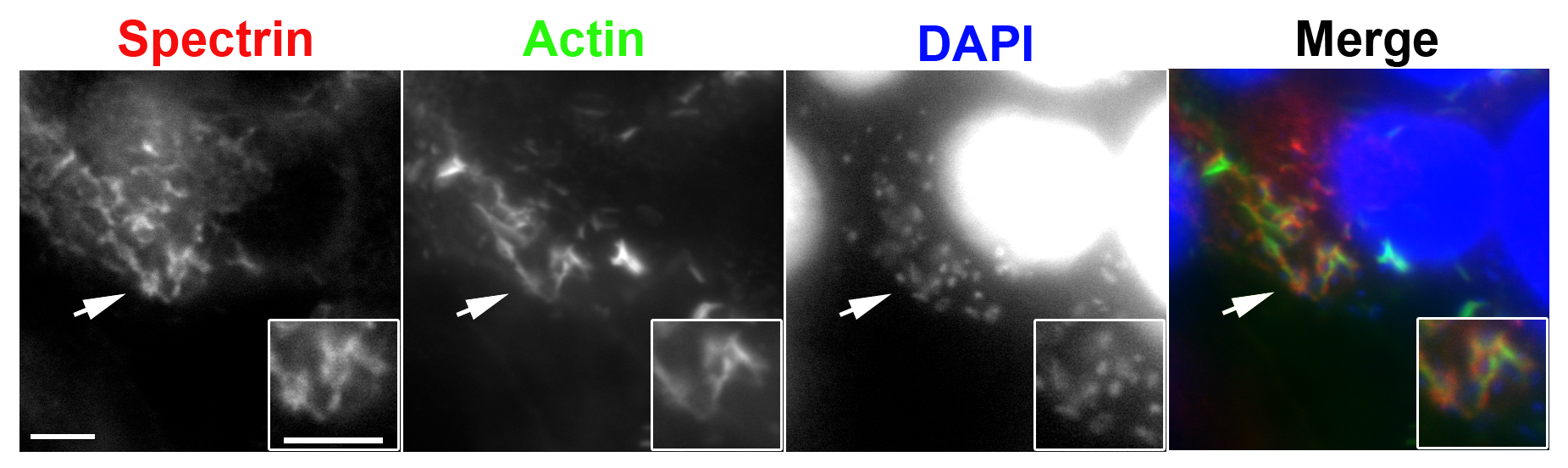

Supplement: Figure S1 — Spectrin is recruited to EPEC pedestals on polarized Caco-2 cells. Polar Caco-2 monolayers were infected with EPEC and stained for spectrin, actin and DAPI. Arrow points to area of actin and spectrin recruitment that is magnified within the inset. Scale bars are 5 µm. (TIF) [file pone.0019940.s001.tif]

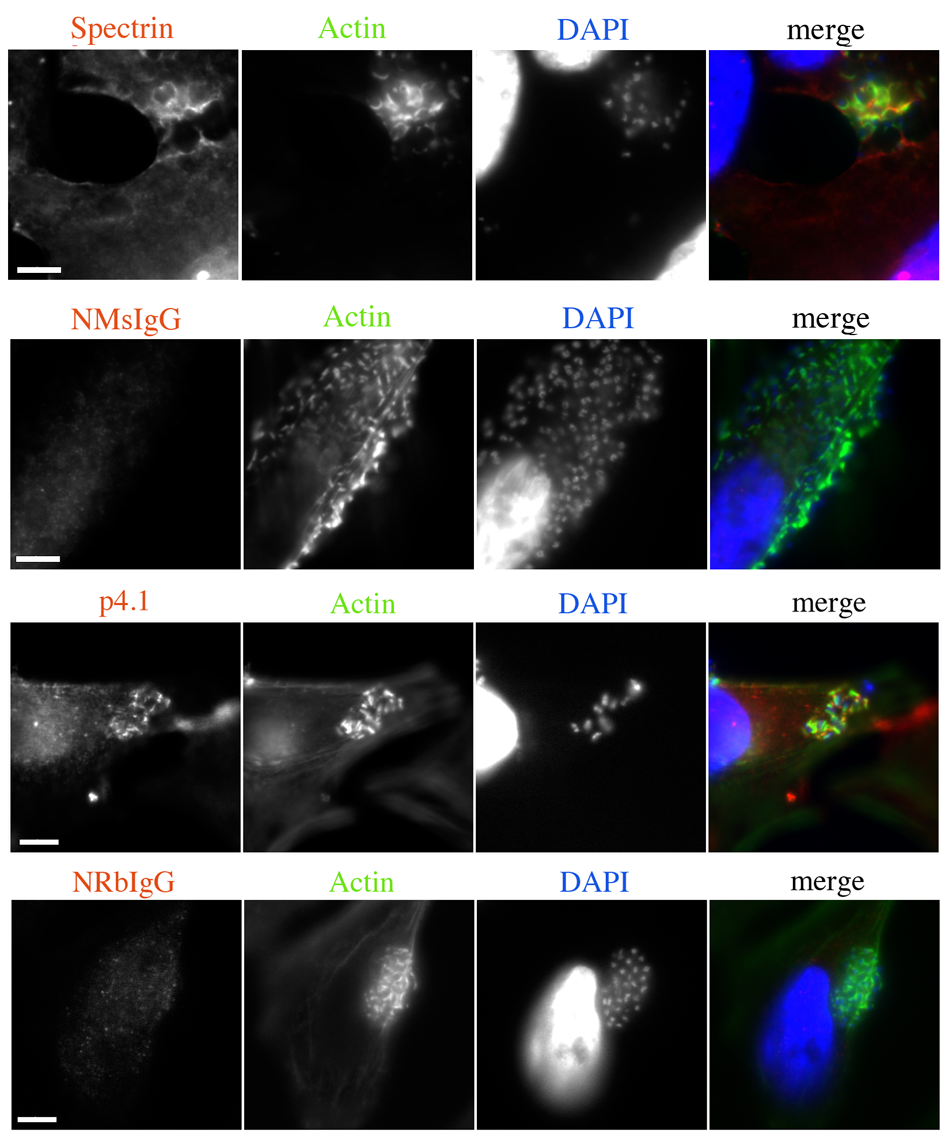

Supplement: Figure S2 — Primary antibody controls show no specific staining at EPEC pedestals. HeLa cells were infected with EPEC for 6 hours. Cells were treated with antibodies specific to spectrin or p4.1 and compared to cells stained with normal mouse IgG (NMsIgG) or normal rabbit IgG (NRbIgG), at identical concentrations to the spectrin and p4.1 antibodies respectively. Primary antibodies or non-specific IgG were co-localized with probes for DAPI and actin to identify attached EPEC and their pedestals. Scale bars are 5 µm. (TIF) [file pone.0019940.s002.tif]

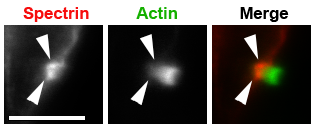

Supplement: Figure S3 — Spectrin localizes to the basal region of EPEC pedestals. HeLa cells were infected with EPEC and stained for spectrin and actin. Arrows indicate a concentration of spectrin at the pedestal but it is not recruited to areas of actin filament concentration. (TIF) [file pone.0019940.s003.tif]

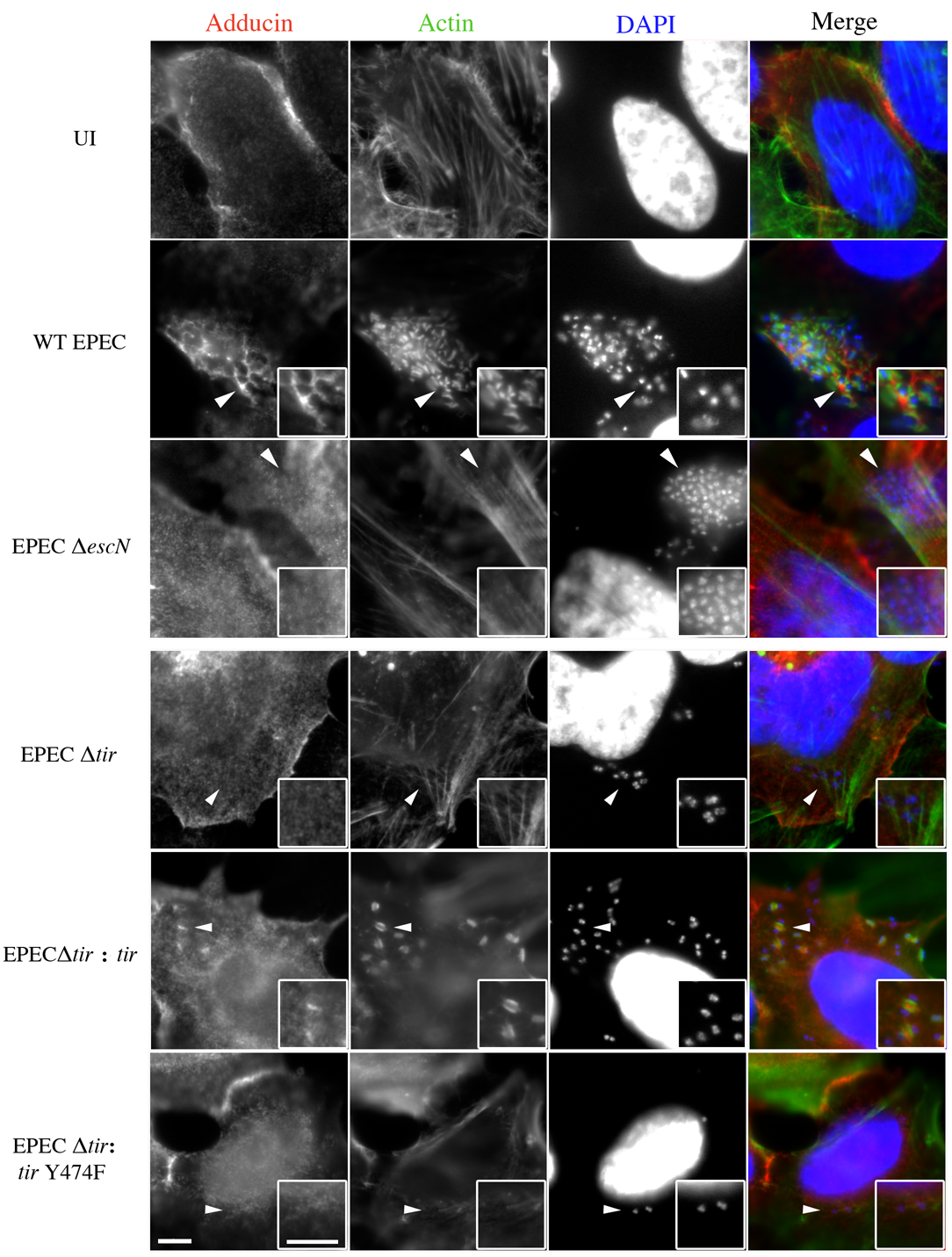

Supplement: Figure S4 — The role of EPEC effectors in adducin recruitment to pedestals. HeLa cells were infected with EPEC or EPEC effector mutants, and immunolocalized with adducin antibodies, as well as actin and DAPI. Arrows indicate areas of interest that are found in the insets. Images examining adducin localization in uninfected (UI) or infections with WT EPEC, EPEC ΔescN, EPEC Δtir, EPEC Δtir:tir, and EPEC Δtir:tirY474F. Scale bars are 5 µm. (TIF) [file pone.0019940.s004.tif]

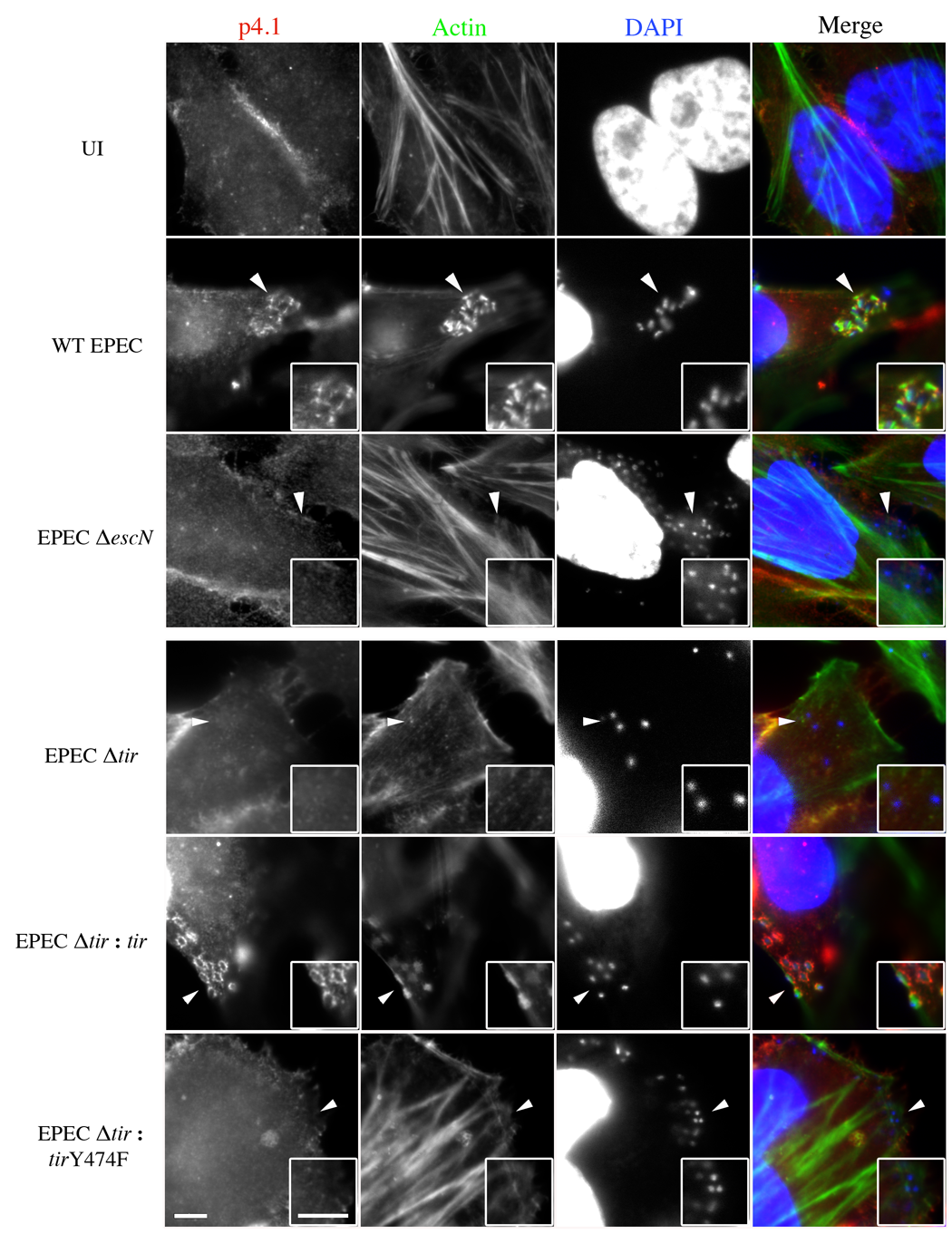

Supplement: Figure S5 — P4.1 recruitment in host cells during EPEC infections. HeLa cells were infected with various EPEC effector mutants and immunolocalized with an antibody targeted against p4.1, as well as probes to actin and DAPI. Arrows indicate areas of interest that are found in the insets. Figure shows immunolocalization of p4.1 during infections with WT EPEC, EPEC ΔescN, EPEC Δtir, EPEC Δtir:tir, EPEC Δtir:tirY474F. Scale bars are 5 µm. (TIF) [file pone.0019940.s005.tif]

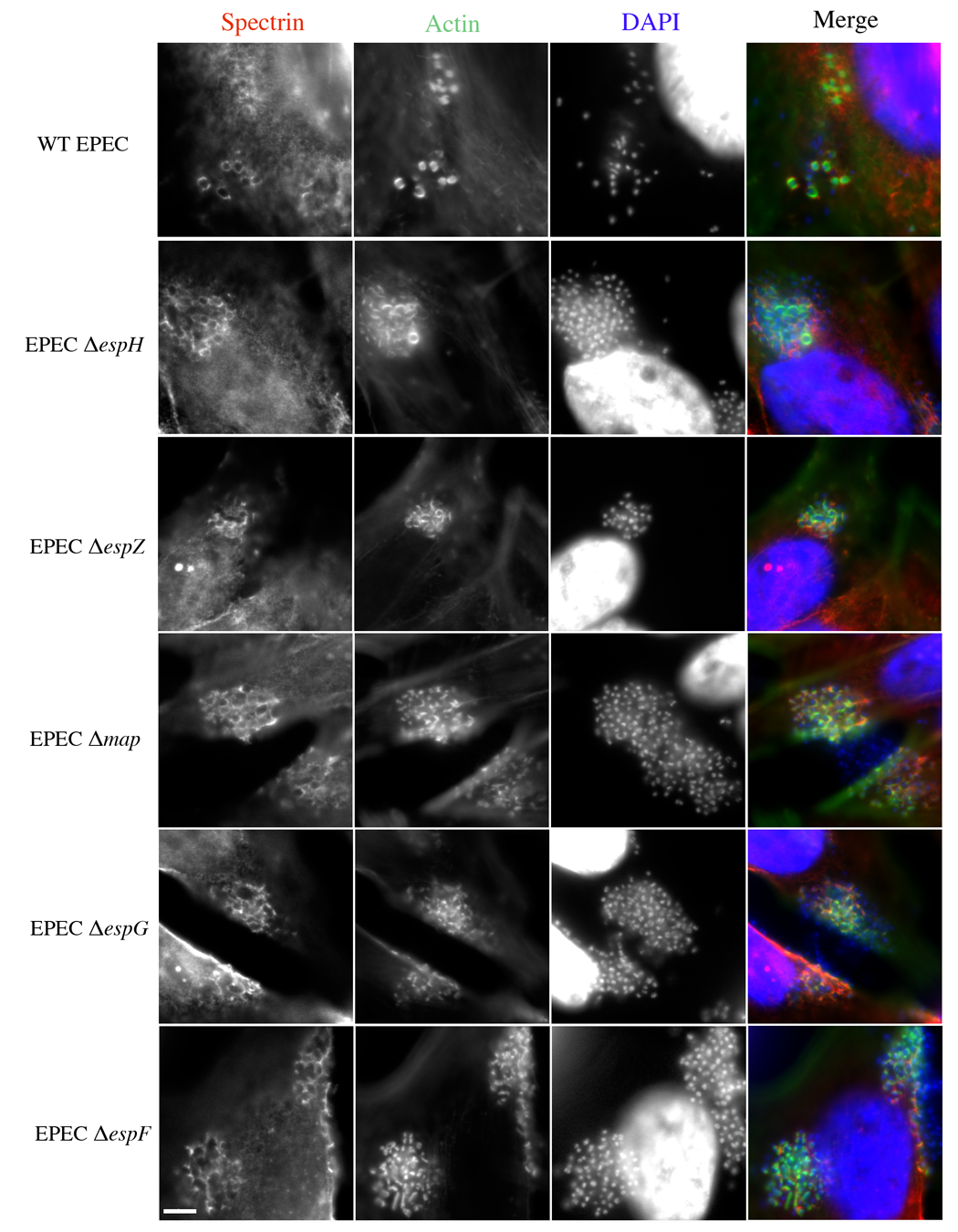

Supplement: Figure S6 — Spectrin recruitment to pedestals generated during other EPEC effector mutants that are also involved in efficient pedestal formation. HeLa cells were infected with EPEC or EPEC effector mutants and immunolocalized with an anti-spectrin antibody and co-localized with actin and DAPI. Figure shows immunolocalization of spectrin during infections with WT EPEC, EPEC ΔespH, EPEC ΔespZ, EPEC Δmap, EPEC ΔespG, EPEC ΔespF. Scale bars are 5 µm. (TIF) [file pone.0019940.s006.tif]

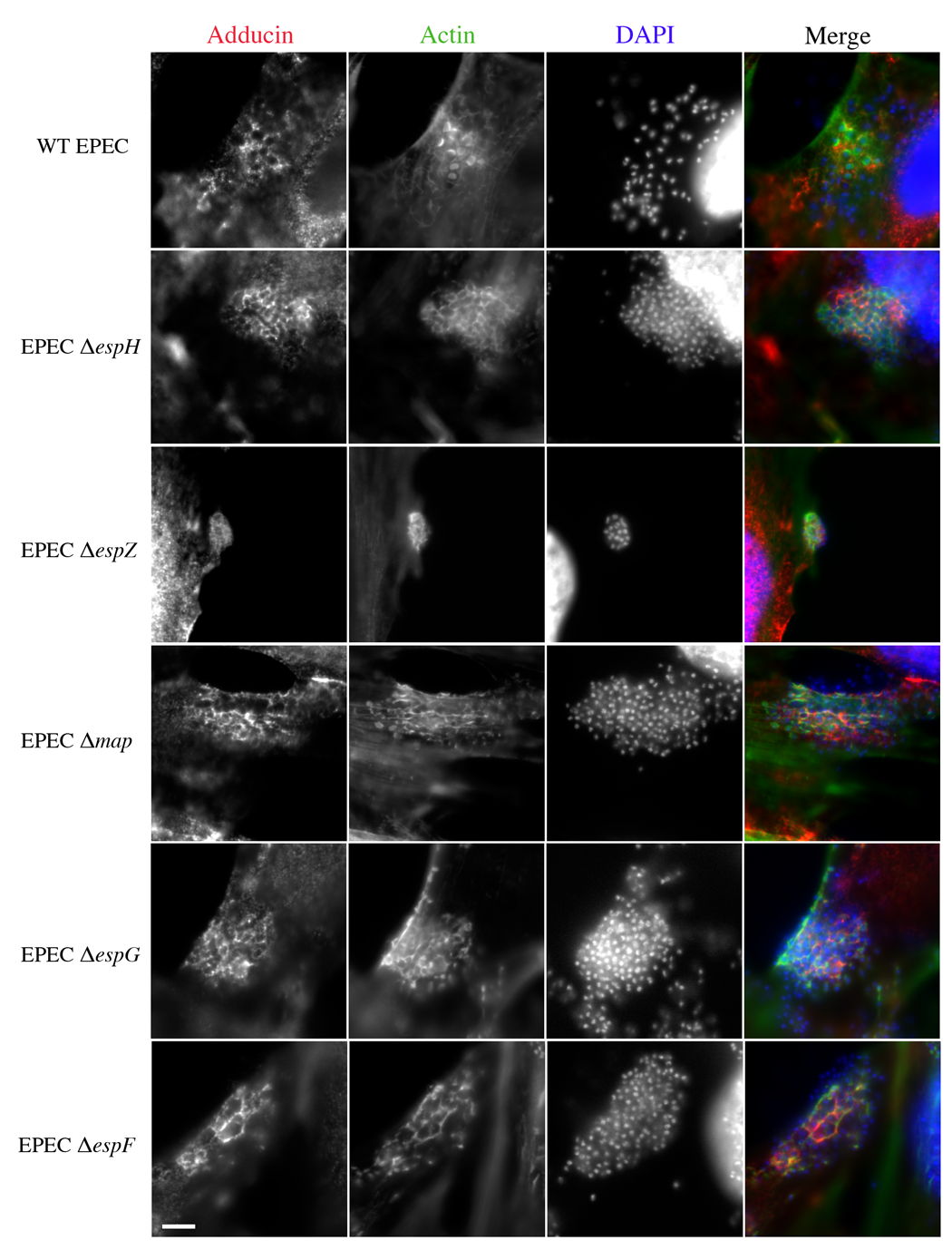

Supplement: Figure S7 — Immunolocalization of adducin, actin and DAPI during infections with EPEC effector mutants on HeLa cells. The figure shows immunolocalization of adducin to pedestals of WT EPEC, EPEC ΔespH, EPEC ΔespZ, EPEC Δmap, EPEC ΔespG, EPEC ΔespF. Scale bars are 5 µm. (TIF) [file pone.0019940.s007.tif]

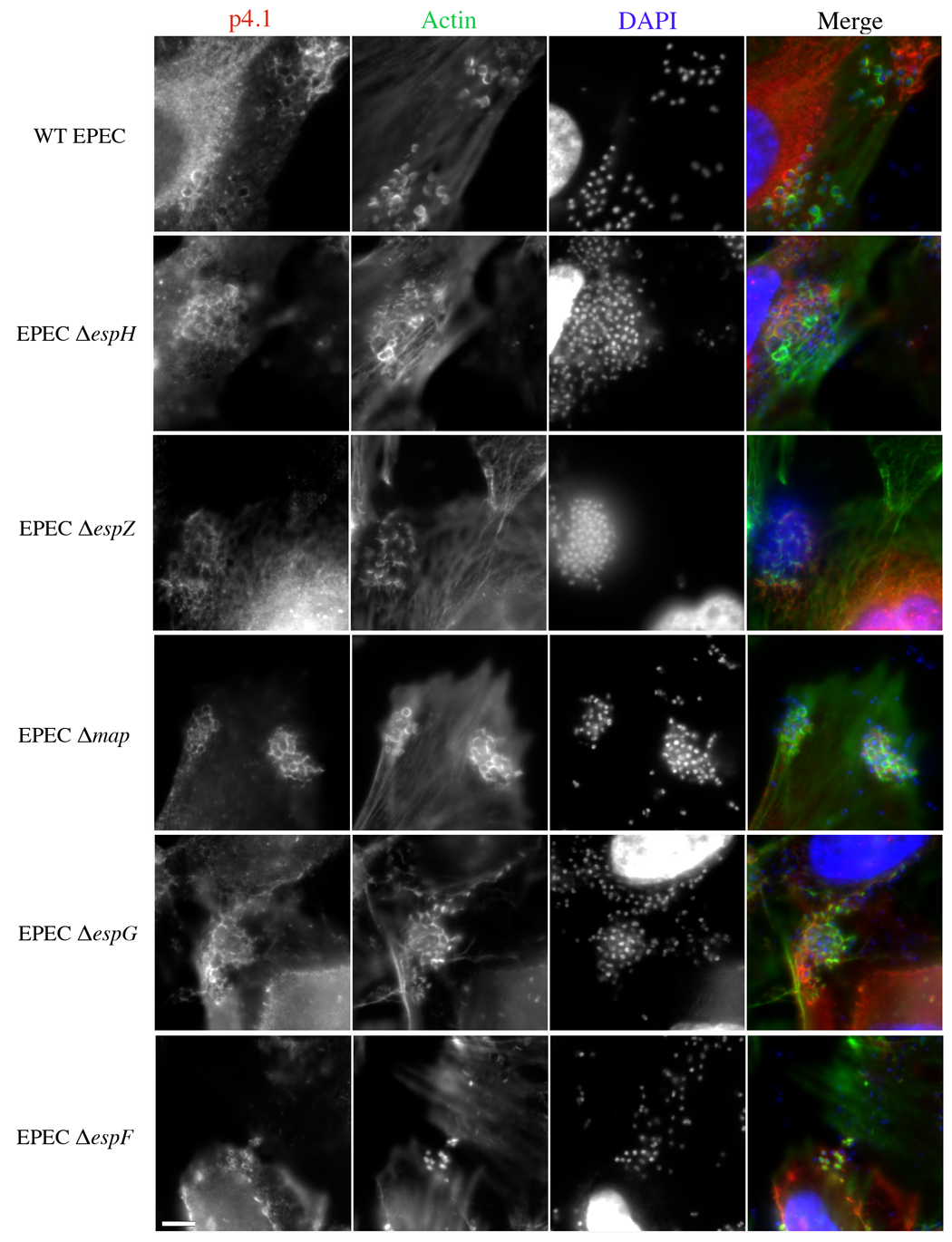

Supplement: Figure S8 — P4.1 actin and DAPI co-localization during infections with EPEC effector mutants. Figure showing recruitment of p4.1 to pedestals of WT EPEC, EPEC ΔespH, EPEC ΔespZ, EPEC Δmap, EPEC ΔespG, EPEC ΔespF. Scale bars are 5 µm. (TIF) [file pone.0019940.s008.tif]

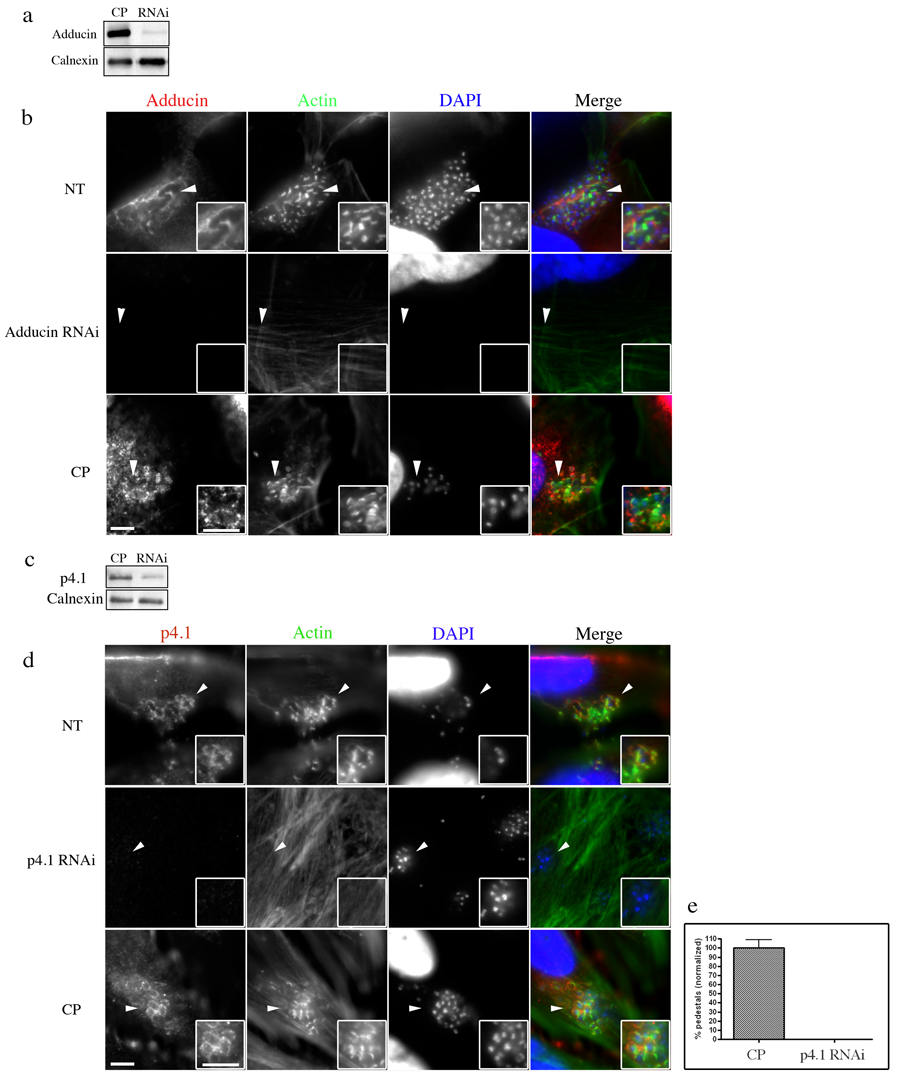

Supplement: Figure S9 — Adducin and p4.1 are crucial for EPEC attachment and pedestal formation repectively. (a) Adducin was knocked-down in host cells. (b) EPEC infected cells were labeled with adducin, actin and DAPI. Bacteria did not attach to adducin RNAi cells, but attached and generated pedestals in cells with no treatment (NT) and control pool (CP) siRNA treated cells. (c) Western blot confirming p4.1 was knocked down using siRNA (RNAi). Cells were infected with wild-type EPEC and pedestals counted. (d) Immunofluorescent images and (e) quantification of the number of bacteria forming pedestals. For each treatment, 3 independent experiments were run; for microscopy counts n = 3, error bars show s.e.m. No stats run due to a complete absence of pedestals generated in infected RNAi samples. Scale bars are 5 µm. (TIF) [file pone.0019940.s009.tif]

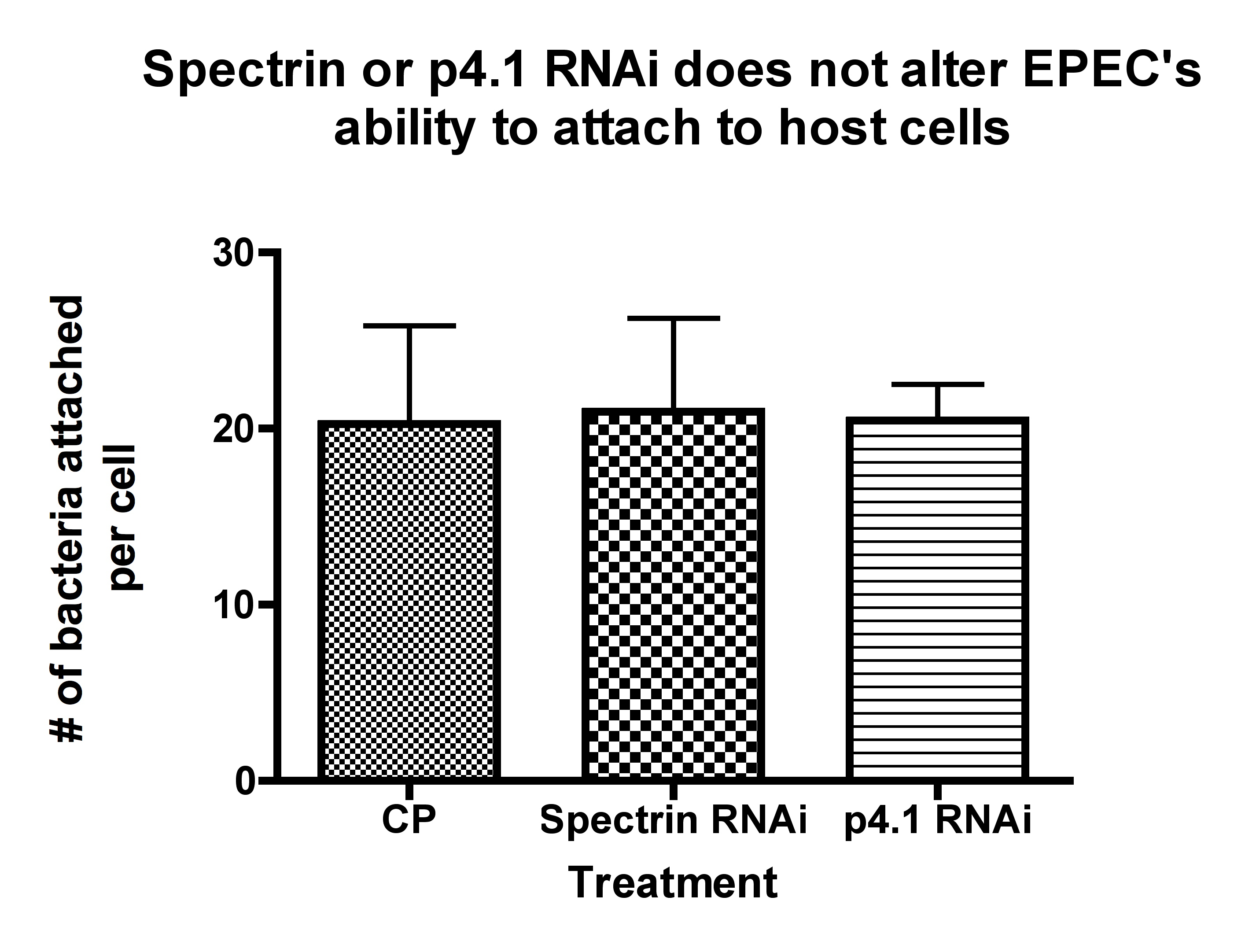

Supplement: Figure S10 — Spectrin or p4.1 knockdowns do not influence the ability of EPEC to attach to the host cell. HeLa cells were transfected with control pool (CP), spectrin or p4.1 siRNA, then infected with EPEC for 6 hours. The average number of bacteria attached to each cell was then counted. Each experiment was run in triplicate (n = 3) and 30 host cells were counted per treatment. The means of each treatment were not statistically significant (P<0.05). Error bars show s.e.m. (TIF) [file pone.0019940.s010.tif]

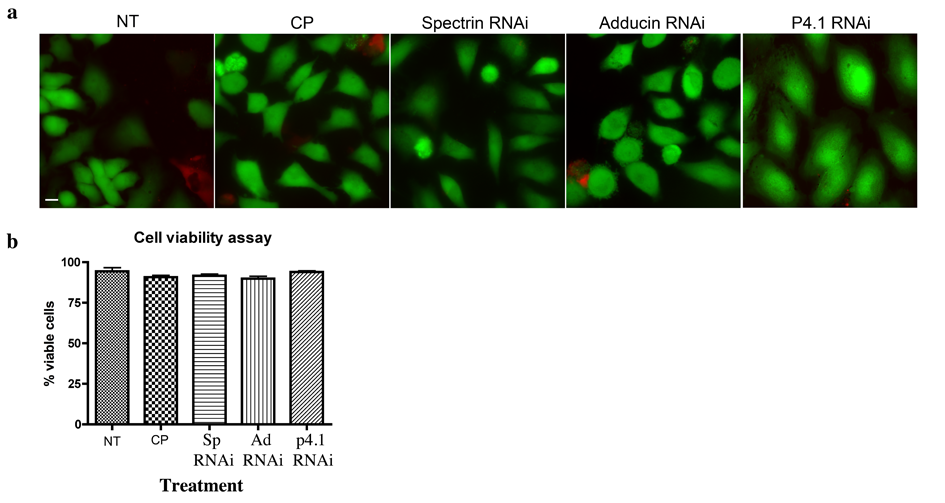

Supplement: Figure S11 — Viability of cells is unaltered by various siRNA treatments. Hela cells were left untreated (NT = no treatment) or treated with control pool (CP), spectrin, p4.1, or adducin siRNA identically to our infection siRNA protocols. (a) The cells were stained with a cell viability probe (Invitrogen). Green cells represent viable cells, red cells represent dead cells. For each treatment, 3 independent experiments were run (n = 3). (b) Total cell viability of each treatment was quantified by counting 200 cells in each sample. The means of each treatment are not statistically significant (P<0.05). Error bars show s.e.m. Scale bar is 5 µm. (TIF) [file pone.0019940.s011.tif]

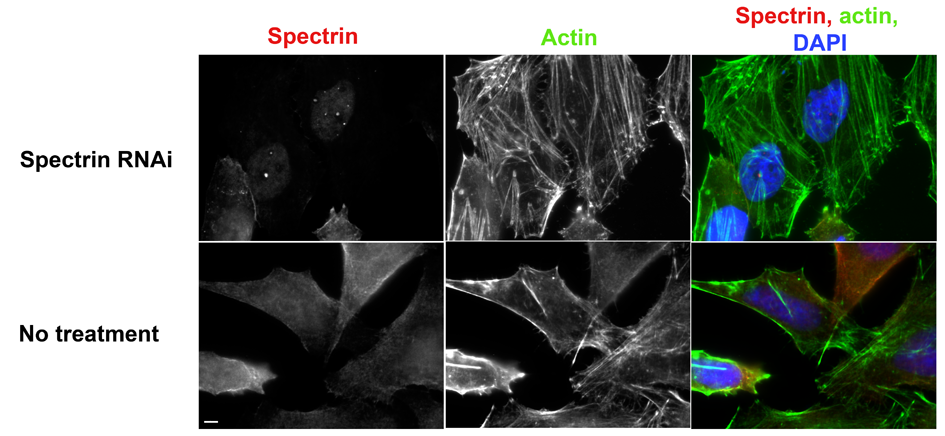

Supplement: Figure S12 — Actin cytoskelton morphology is unaltered during spectrin knockdown. HeLa cells were treated with spectrin siRNA for 48 hours. Cells were stained for actin, spectrin and DAPI. The actin cytoskeleton morphology appears normal, with characteristic cortical actin and stress fibers present in the cells. Scale bar is 5 µm. (TIF) [file pone.0019940.s012.tif]

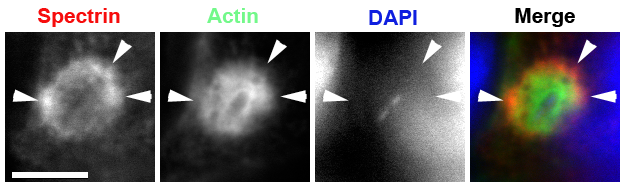

Supplement: Figure S13 — Spectrin is recruited to membrane ruffles during S. Typhimurium invasion of Caco-2 cell monolayers. Polarized Caco-2 cells were infected with S. Typhimurium for 15 minutes and immunolocalized with spectrin, actin and DAPI. Arrows indicated regions where spectrin is present peripheral to actin at the membrane ruffles. Scale bar is 5 µm. (TIF) [file pone.0019940.s013.tif]

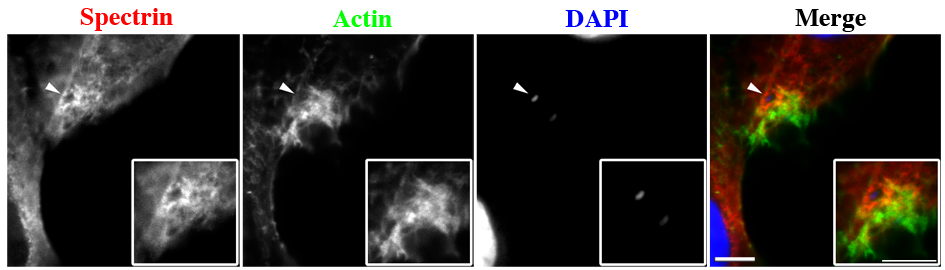

Supplement: Figure S14 — Spectrin is present at regions of S. Typhimurium membrane ruffles independent of actin. Immunolocalization of spectrin, actin and DAPI during infection of HeLa cells with S. Typhimurium. Arrowhead and inset identify a site of invasion, demonstrating spectrin recruitment at site of bacterial invasion that are independent of actin in certain regions. Scale bars are 5 µm. (TIF) [file pone.0019940.s014.tif]

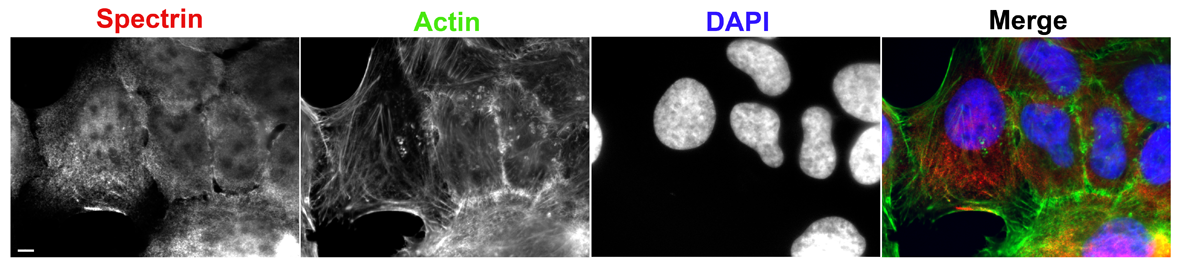

Supplement: Figure S15 — Examples of actin cytoskeletal network in regions where spectrin is absent in uninfected cells. Uninfected HeLa cells were stained for actin, spectrin, and DAPI. Stress fibers and the cell cortex are present with actin in the absence of spectrin. Scale bar is 5 µm. (TIF) [file pone.0019940.s015.tif]

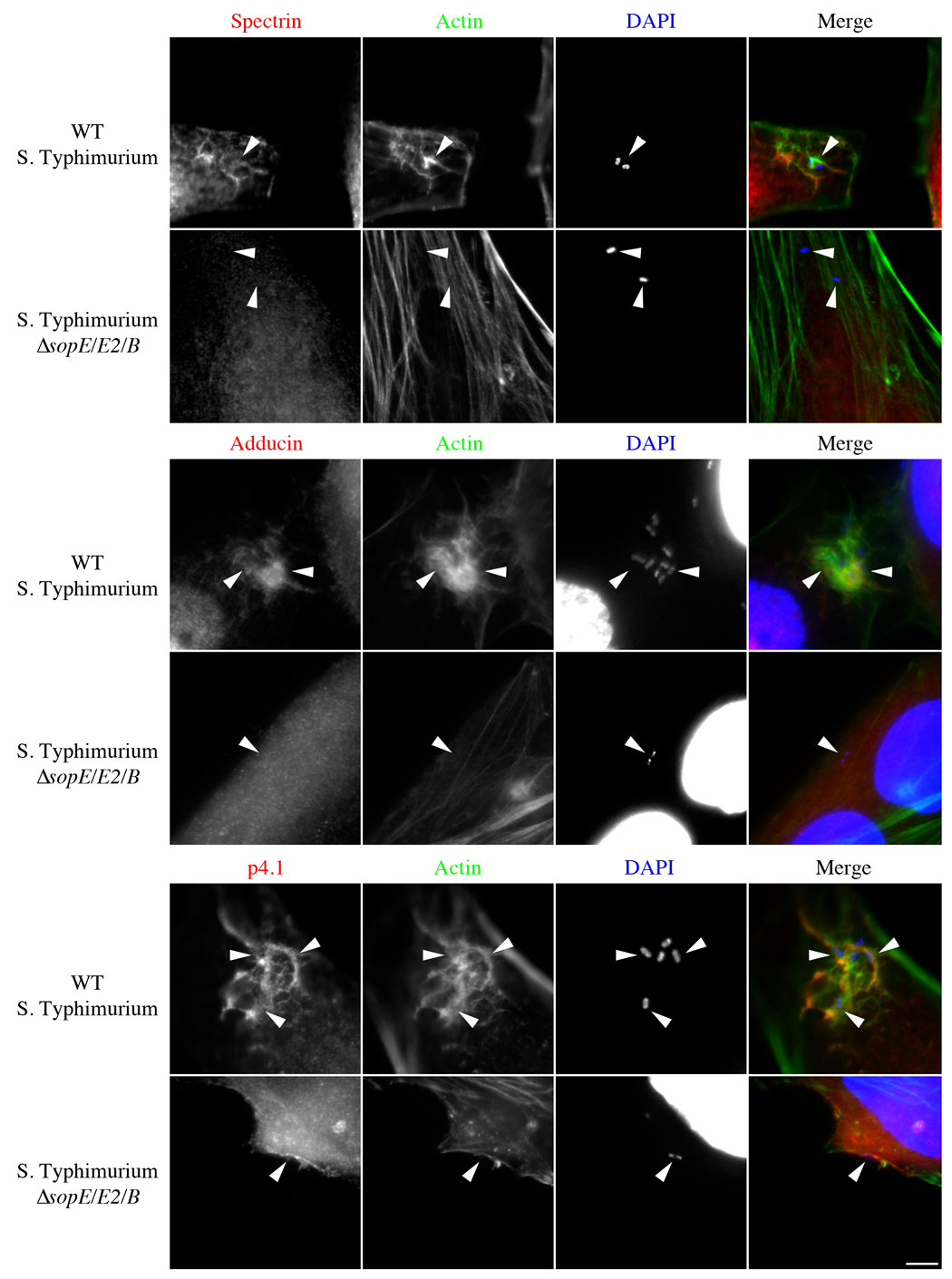

Supplement: Figure S16 — Membrane ruffles are needed for spectrin cytoskeletal protein recruitment. Immunofluorescence images of spectrin, adducin and p4.1 with DAPI and actin during infection with Salmonella ΔsopE/E2/B mutant compared to WT Salmonella. Arrows indicate areas of interest. S. Typhimurium ΔsopE/E2/B did not generate membrane ruffles and did not recruit spectrin cytoskeletal proteins. Scale bar is 5 µm. (TIF) [file pone.0019940.s016.tif]

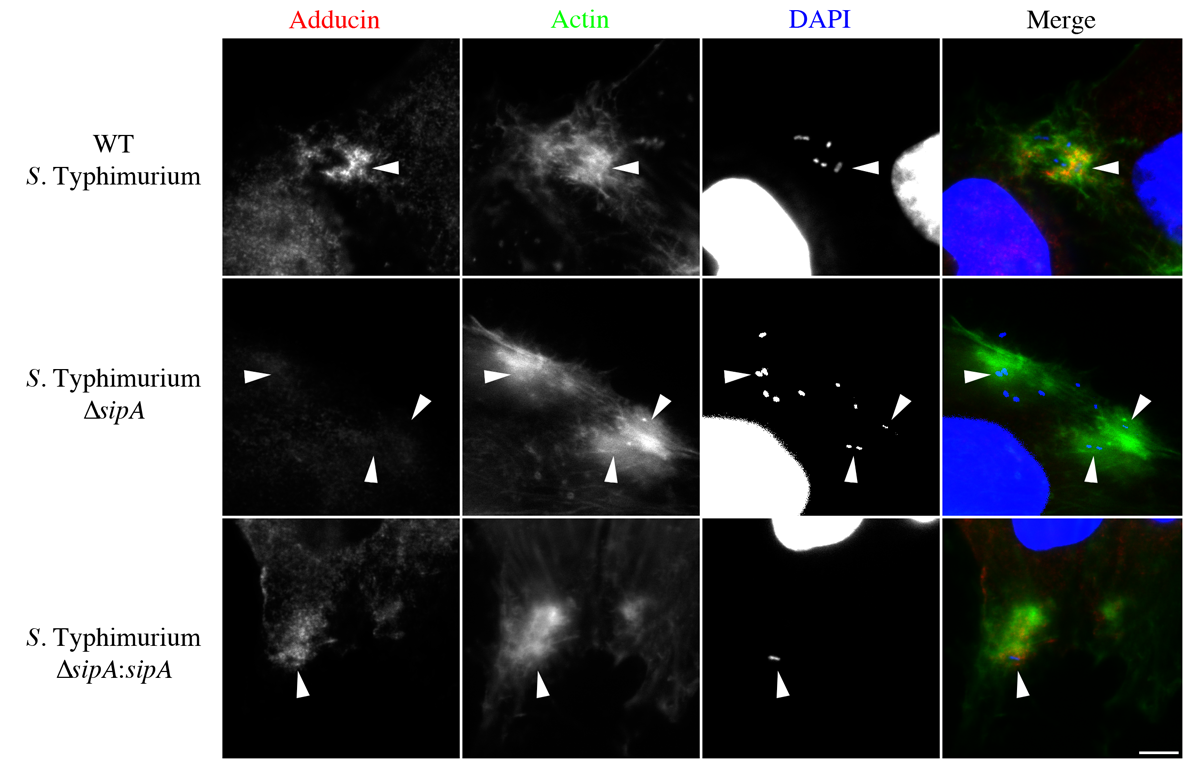

Supplement: Figure S17 — SipA is required for adducin recruitment to membrane ruffles. Adducin was immunolocalized during S. Typhimurium ΔsipA infections. Images show a lack of adducin recruitment to invasion sites with actin-rich membrane ruffling on HeLa cells infected with S. Typhimurium ΔsipA. Complemented S. Typhimurium ΔsipA:sipA rescued the wild-type phenotype. Scale bar is 5 µm. (TIF) [file pone.0019940.s017.tif]

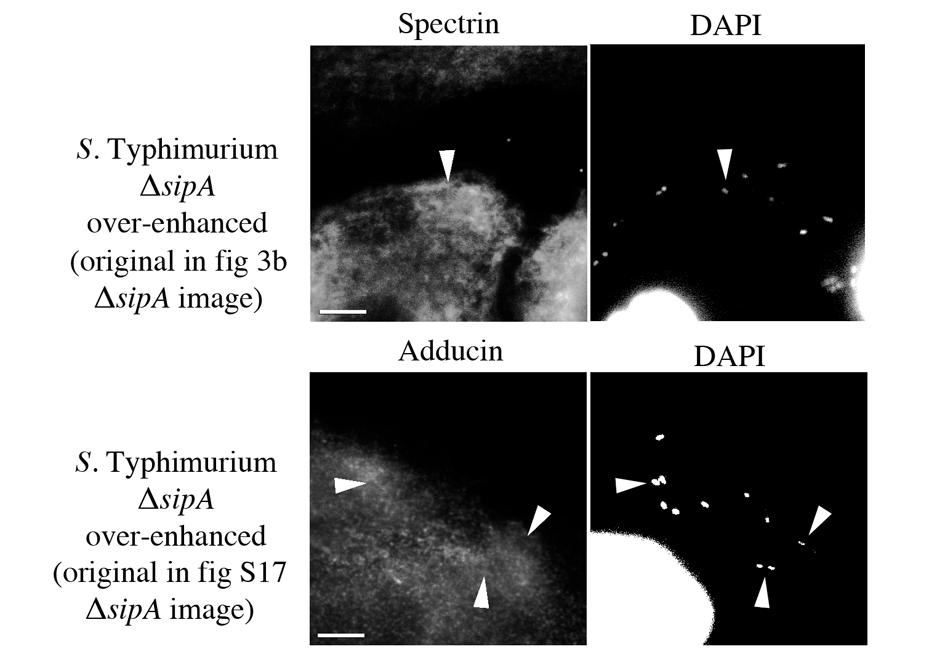

Supplement: Figure S18 — S. Typhimurium ΔsipA infections with over-enhanced images to indicate that a cell was present in the S. Typhimurium ΔsipA panels in figures 3b and S15. Background spectrin and adducin host cell levels are presented. Scale bars are 5 µm. (TIF) [file pone.0019940.s018.tif]

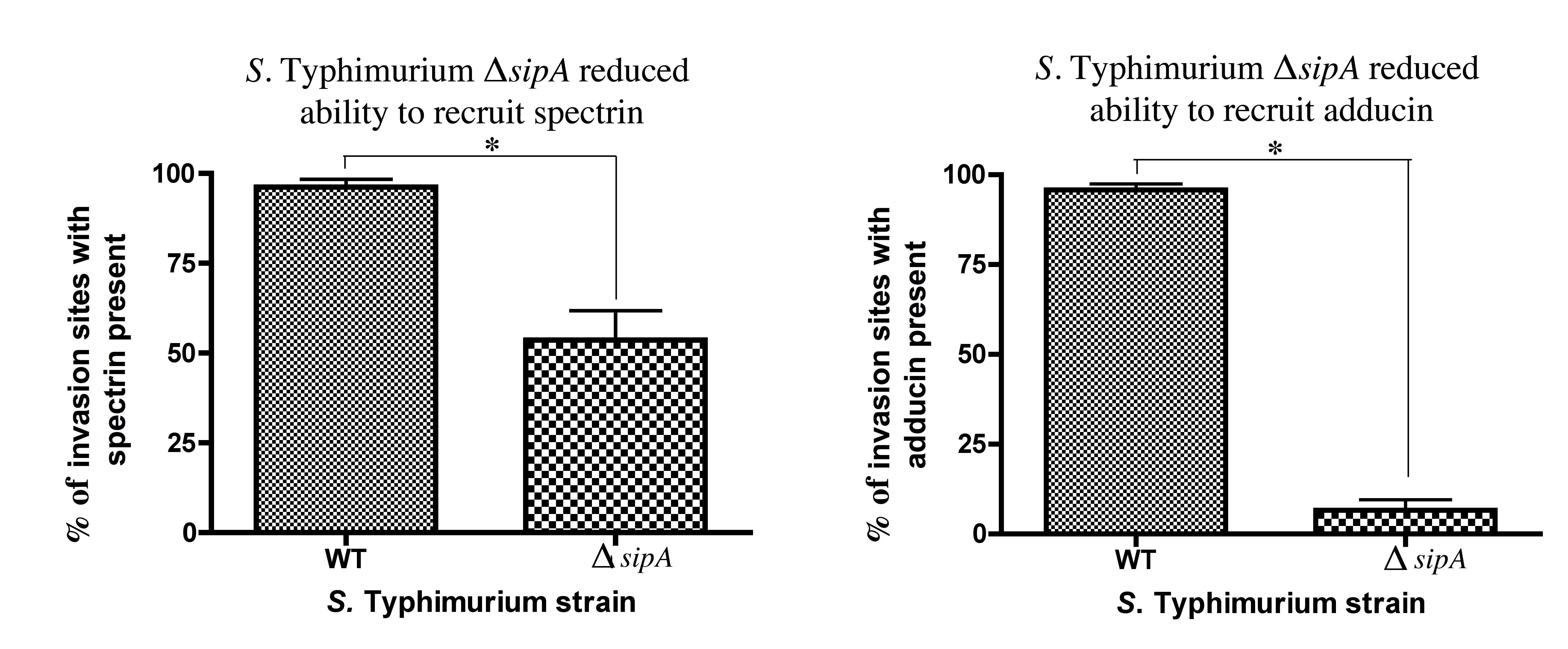

Supplement: Figure S19 — S. Typhimurium ΔsipA infections showed reduced ability to recruit spectrin and adducin. Quantification of spectrin and adducin recruitment to sites of S. Typhimurium ΔsipA invasion as compared to WT S. Typhimurium invasion. Invasion sites were identified by actin-rich membrane ruffles around attached bacteria, then observed for spectrin or adducin recruitment to those sites. Each experiment was performed in triplicate (n = 3), counting 100 actin-based invasion sites. The means of the WT versus ΔsipA infection are significant (P<0.0001). Error bars show s.e.m. (TIF) [file pone.0019940.s019.tif]

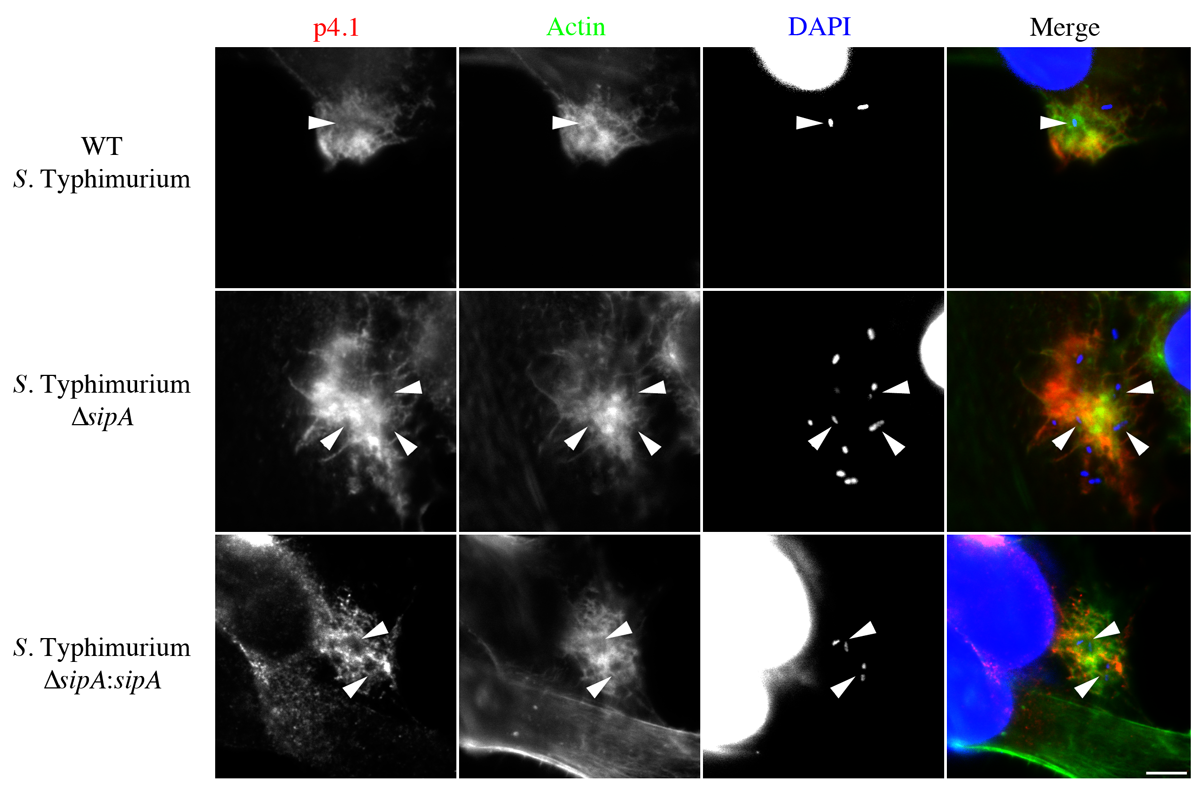

Supplement: Figure S20 — P4.1 accumulation is unaltered by a mutation in sipA. S. Typhimurium ΔsipA infected HeLa cells recruited protein 4.1 to membrane ruffles during invasion. P4.1 localization was maintained during S. Typhimurium wild-type, ΔsipA, or ΔsipA:sipA infected cells. Scale bar is 5 µm. (TIF) [file pone.0019940.s020.tif]

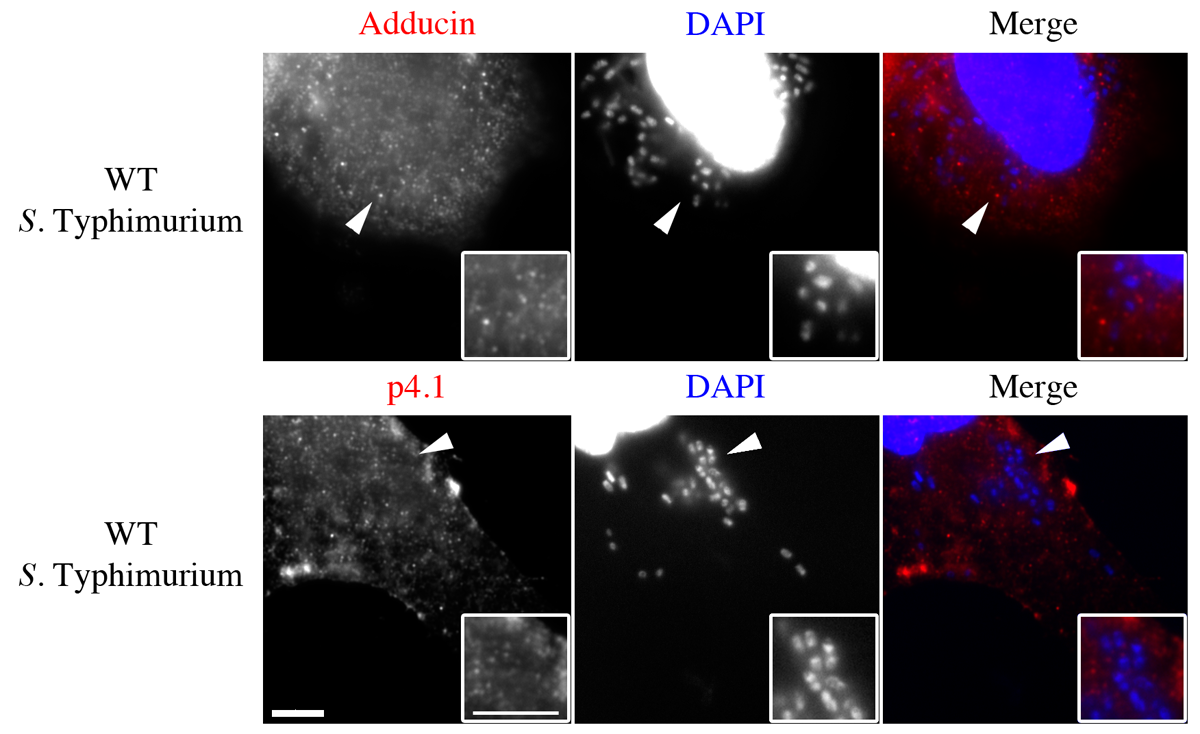

Supplement: Figure S21 — Adducin and p4.1 are not recruited to SCV's. S. Typhimurium infected HeLa cells were immunolocalized with anti-adducin or anti-p4.1 antibodies together with DAPI at 90 minutes post infection. No accumulation of adducin or protein 4.1 was detected. Areas of interest are indicated by arrowheads and highlighted in insets. Scale bars are 5 µm. (TIF) [file pone.0019940.s021.tif]

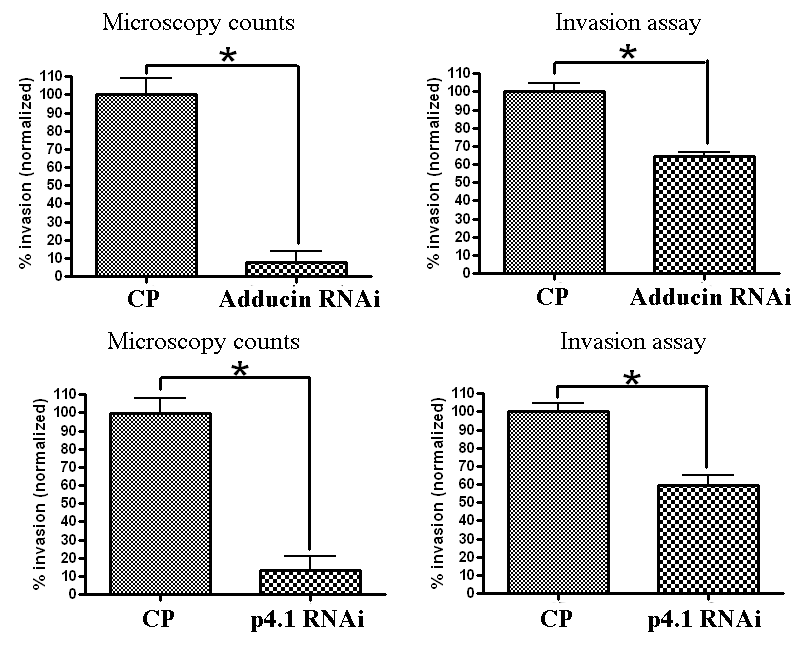

Supplement: Figure S22 — Adducin and p4.1 are crucial for efficient S. Typhimurium invasion. Adducin and p4.1 were individually knocked down in HeLa cells and infected with wild-type S. Typhimurium. Samples were assayed my microscopic counts and invasion assays and compared to non-targeting control pools (CP) of siRNAs. For each treatment, 3 independent experiments were run; for microscopy counts n = 3, error bars show s.e.m, *P<0.0001 for all statistics. Microscopy counts focused on cells with complete knockdown, counting total number of internalized bacteria. Invasion assays involved typical gentamicin survival assay. (TIF) [file pone.0019940.s022.tif]

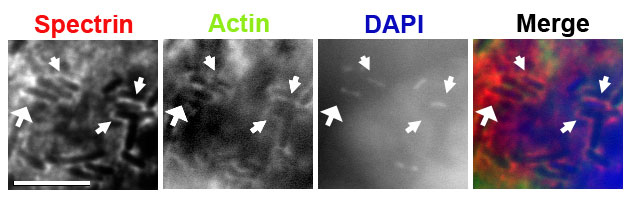

Supplement: Figure S23 — Spectrin is recruited to L. monocytogenes at the initial stages of comet tail formation in polar Caco-2 cells. Caco-2 monolayers were infected with L. monocytogenes for 30 minutes and stained for spectrin, actin and DAPI. Large arrows show areas where spectrin was recruited to bacterial membranes without actin, while small arrows show co-localization of spectrin and actin at the bacterial membrane. Scale bar is 5 µm. (TIF) [file pone.0019940.s023.tif]

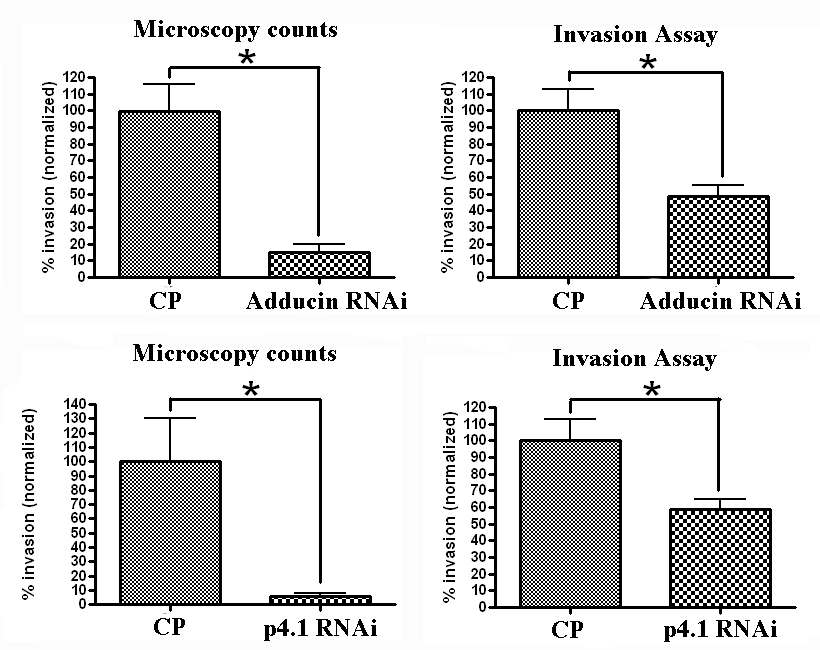

Supplement: Figure S24 — Adducin and p4.1 are crucial for efficient Listeria invasion. Adducin, p4.1 or control pool (CP) non-targetting RNAi treated HeLa cells were infected with L. monocytogenes and quantified by microscopy and invasion assays. For each treatment, 3 independent experiments were run; for microscopy counts n = 3, error bars show s.e.m, *P<0.0001 for all statistics. Microscopy counts focused on cells with complete knockdown, counting total number of internalized bacteria. Invasion assays involved typical gentamicin survival assay. (TIF) [file pone.0019940.s024.tif]

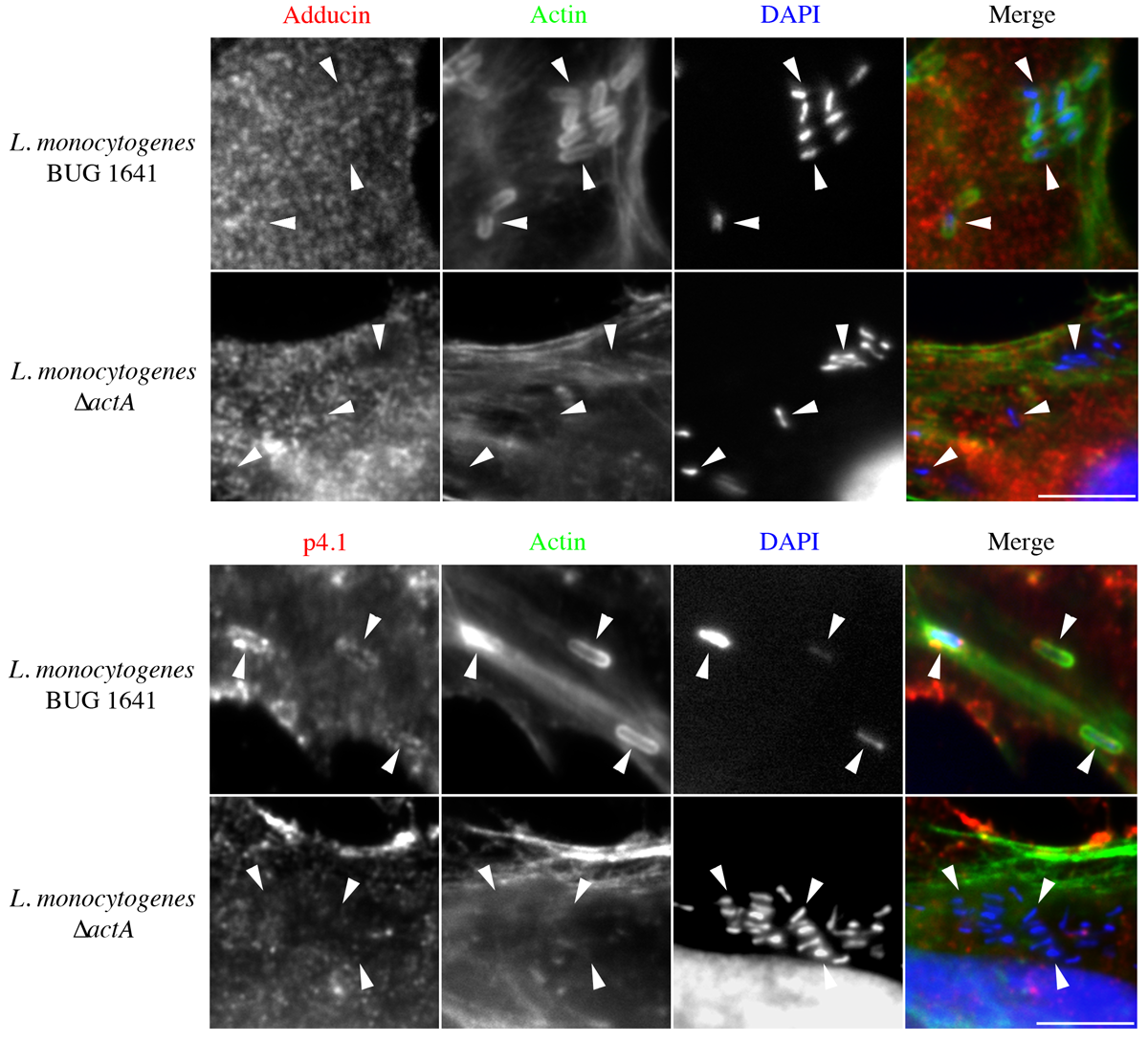

Supplement: Figure S25 — Act A is needed for p4.1 recruitment to sites of L. monocytogenes comet tail formation but does not influence the lack of adducin recruitment. Immunoflourescence images depicting p4.1, adducin, actin and DAPI at 90 minutes post infection. L. monocytogenes ΔactA infections show no actin, adducin or p4.1 recruitment, whereas wild-type L. monocytogenes (BUG 1641) containing actA recruits actin and p4.1, but not adducin. Scale bars are 5 µm. (TIF) [file pone.0019940.s025.tif]

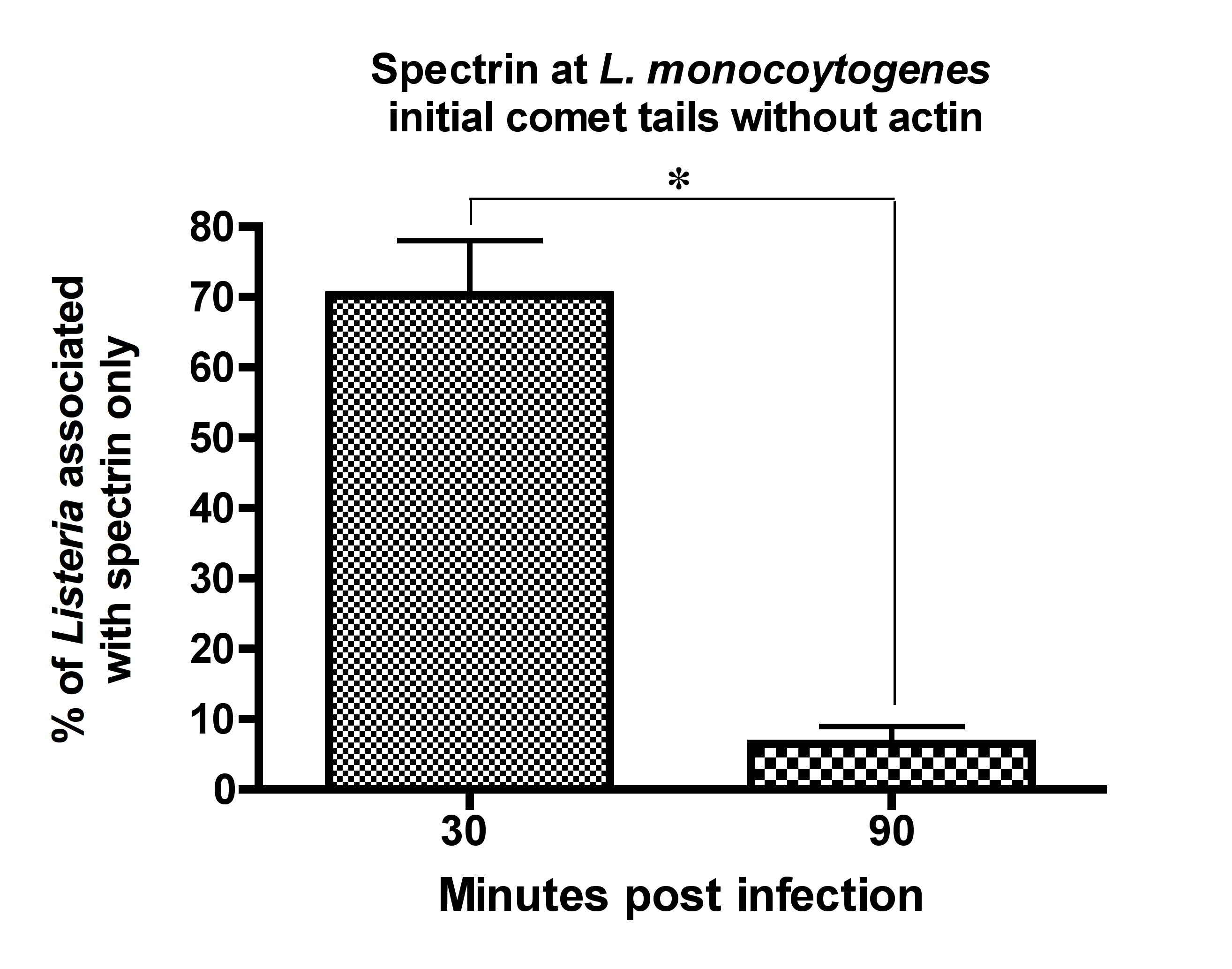

Supplement: Figure S26 — Quantification of spectrin localized at internalized L. monocytogenes in the absence of actin. HeLa cells infected for 30 or 90 minutes with L. monocytogenes were immunolocalized with spectrin, actin and DAPI. Internalized bacteria associated with spectrin were quantified and compared to bacteria associated with both spectrin and actin. The graph depicts the percentage of bacteria associated with spectrin alone at various time points. 100 internalized bacteria were counted per experiment (n = 3). Each experiment was run in triplicate. The means of the two data sets are significantly different (P<0.05). Error bars show s.e.m. (TIF) [file pone.0019940.s026.tif]

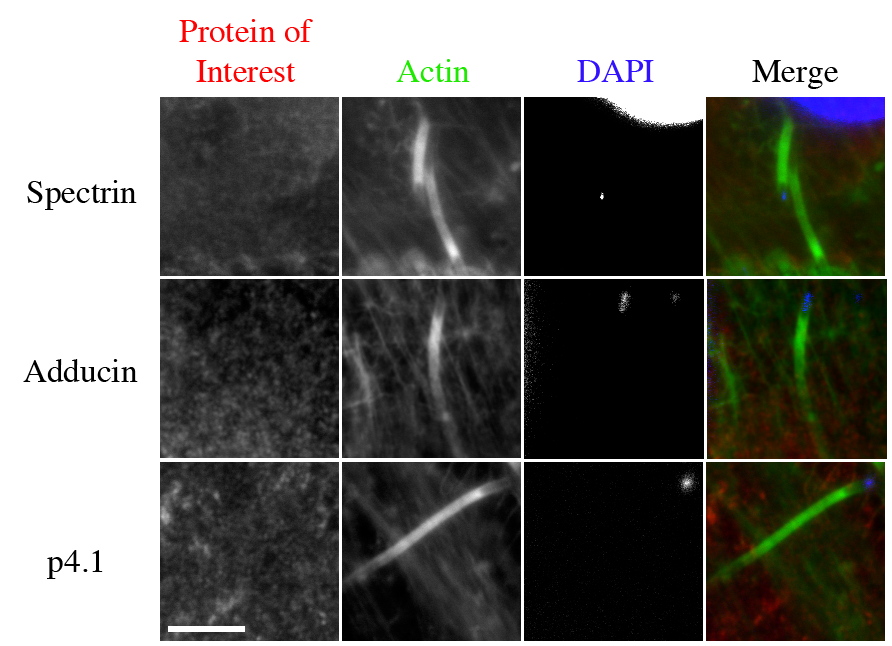

Supplement: Figure S27 — Spectin cytoskeletal components are absent from established L. monocytogenes comet tails. Spectin, adducin, and p4.1 together with actin and DAPI were labeled on L. monocytogenes infected HeLa cells 3 hours post infection. None of the spectrin cytoskeletal proteins were recruited to comet tails. Scale bars are 5 µm. (TIF) [file pone.0019940.s027.tif]
